# Recent advance in bioactive hydrogels for repairing spinal cord injury: material design, biofunctional regulation, and applications

**DOI:** 10.1186/s12951-023-01996-y

**Published:** 2023-07-24

**Authors:** Zhengang Sun, Danzhu Zhu, Hong Zhao, Jia Liu, Peng He, Xin Luan, Huiqiang Hu, Xuanfen Zhang, Gang Wei, Yongming Xi

**Affiliations:** 1 Department of Spinal Surgery, Affiliated Hospital of Qingdao University, Qingdao, 266071 People’s Republic of China; 2 Department of Spinal Surgery, Huangdao Central Hospital, Affiliated Hospital of Qingdao University, Qingdao, 266071 China; 3 College of Chemistry and Chemical Engineering, Qingdao University, Qingdao, 266071 People’s Republic of China; 4 The Department of Plastic Surgery, Lanzhou University Second Hospital, Lanzhou, 730030 People’s Republic of China

**Keywords:** Spinal cord injury, Hydrogels, Functional regulation, Bioactivity, Biomedical engineering

## Abstract

Functional hydrogels show potential application in repairing spinal cord injury (SCI) due to their unique chemical, physical, and biological properties and functions. In this comprehensive review, we present recent advance in the material design, functional regulation, and SCI repair applications of bioactive hydrogels. Different from previously released reviews on hydrogels and three-dimensional scaffolds for the SCI repair, this work focuses on the strategies for material design and biologically functional regulation of hydrogels, specifically aiming to show how these significant efforts can promoting the repairing performance of SCI. We demonstrate various methods and techniques for the fabrication of bioactive hydrogels with the biological components such as DNA, proteins, peptides, biomass polysaccharides, and biopolymers to obtain unique biological properties of hydrogels, including the cell biocompatibility, self-healing, anti-bacterial activity, injectability, bio-adhesion, bio-degradation, and other multi-functions for repairing SCI. The functional regulation of bioactive hydrogels with drugs/growth factors, polymers, nanoparticles, one-dimensional materials, and two-dimensional materials for highly effective treating SCI are introduced and discussed in detail. This work shows new viewpoints and ideas on the design and synthesis of bioactive hydrogels with the state-of-the-art knowledges of materials science and nanotechnology, and will bridge the connection of materials science and biomedicine, and further inspire clinical potential of bioactive hydrogels in biomedical fields.

## Introduction

Spinal cord injury (SCI) is a kind of spinal surgical disease with serious conditions and poor prognosis. The annual incidence is about 10.4 ~ 83.0/million, which has high disability rate and brings heavy economic burden to the families of patients and societies [1]. Traditional methods, including the hormone shock, surgical decompression, spinal fixation, and rehabilitation, have not shown satisfied performance for treating SCI until now, and there is no successful clinical treatment to stimulate the regeneration of human central nervous system (CNS) [2]. Therefore, how to promote the recovery of the nerve function after SCI is a challenging topic for both foundmental and clinical studies currently.

Using the characteristics of neural stem cells (NSCs) such as the self-update and multi-functional differentiation, clinical applications with adding functional nerve cells have been carried out by inducing endogenous NSCs or exogenous NSCs to treat SCI [3]. However, the local inflammatory microenvironment after the SCI is an important factor to affect the cell behavior [4], and therefore it is particularly important to construct a suitable microenvironment to promote the survival, proliferation, and differentiation of endogenous stem cells so as to promote the regeneration of injured spinal cord [5]. A lot of controllable drug release systems that can support the regeneration of stem cells and the delivery a variety of bioactive factors or drugs to construct a microenvironment that suitable for the CNS regeneration have been developed previously [6, 7], which are of great significance in biomedicine and tissue engineering.

In the pre-clinical SCI treatment, hydrogels have been not only used to promote the tissue repair, but also served as bioactive carriers (cells, drugs or bioactive molecules) for local treatment [8, 9]. Clinically, the condition of SCI is very complicated due to different size, shape, and injury degree [10]. In complex clinical cases, surgical manipulation of the spinal cord by implanting a preformed stent or drug delivery device may result in further damage to the spinal cord tissues [11]. Therefore, targeted injecting hydrogels to the SCI sites is very consistent with clinical personalized therapy. After the injection, hydrogels can well combine with the SCI tissue, slowly release stem cells/drugs/bioactive molecules, and show special functions, such as electrical conductivity, anti-inflammatory, adhesion, absorbability, temperature degeneration, and self-healing [12, 13], making hydrogels attractive materials for the SCI repair and regeneration. However, how to prepare multifunctional hydrogels with injectable, anti-inflammatory, conductive, adhesive, absorbable, thermotropic, and self-healing properties for the SCI repair is a great challenge.

Hydrogels have three-dimensional (3D) porous structures with high ater-concent constructing by physical connection or chemical cross-linking. According to the distance between entanglements, hydrogels can be divided into three types, including macroporous, microporous, and non-porous. After resembling the extracellular matrix (ECM), hydrogels can mimic natural human tissues [14]. Therefore, multifunctional hydrogels have high therapeutic potential for the treatment of SCI, and their clinical applications in the delivery of stem cells, drugs, or bioactive molecules are promising [15]. In addition, the transfer of biomaterials is thought to be a more effective alternative strategy to mediate the NSC transplantation. The loading of stem cells, drugs, or different bioactive growth factors (GFs) to hydrogels could promote the functions of ECM, which can achieve the survival, proliferation, and differentiation of transplanted stem cells into nerve cells [16]. A good delivery system can greatly improve the therapeutic effectiveness of stem cells, drugs and different bioactive substances. The neural tissue engineering of multifunctional hydrogels in combination with stem cells, drugs, or different bioactive factors provides a promising strategy for the recovery of SCI [17, 18]. However, due to the limitations of multifunctional hydrogels, such as the amount of loaded stem cells, the number of bioactive molecules, and the limitations of functional transformation, the utilization of functional hydrogels to load stem cells and transmit a variety of different substances or bioactive factors at the same time is still a challenge.

Several important reviews on treating SCI using hydrogels have been released previously. For instance, Wang et al*.* summarized the pathophysiology and clinical manifestation of SCI [19]. In their work, the composition of polymer hydrogels, the cross-linking method, the treatment strategies, and the effects of injected hydrogels on the SCI repair have been introduced and discussed. Walsh et al*.* described the link between the ability of a successful delivered cells or bioactive molecules and their immune response, introduced the latest advances in the treatment of SCI by immune agents, and demonstrated both physical and chemical properties of hydrogels [14]. Silva and co-workers reviewed the advance of hydrogel-based delivery systems for repairing SCI, in which the characteristics of the flow of hydrogels, the size of the mesh, the expansion, degradation, gel temperature, and surface charge on treating SCI have been introduced and analyzed in detail [20]. Peng and co-workers summarized the current status of various hydrogel-based delivery systems that used for the treatment of secondary SCI, and also discussed the functional modification of these hydrogels in order to obtain better therapeutic results [21]. However, the above-mentioned reviews did not explain clearly the effects of the material design and the regulation of hydrogel functions and biological properties on the treating efficiency of hydrogels toward SCI. We believe the regulation of the bioactivity and bio-properties of hydrogels plays great importance for promoting the applications of hydrogels in repairing SCI, and there is still some space that could be filled in to address the promising applications of hydrogels in the SCI repair.

**Scheme 1.**
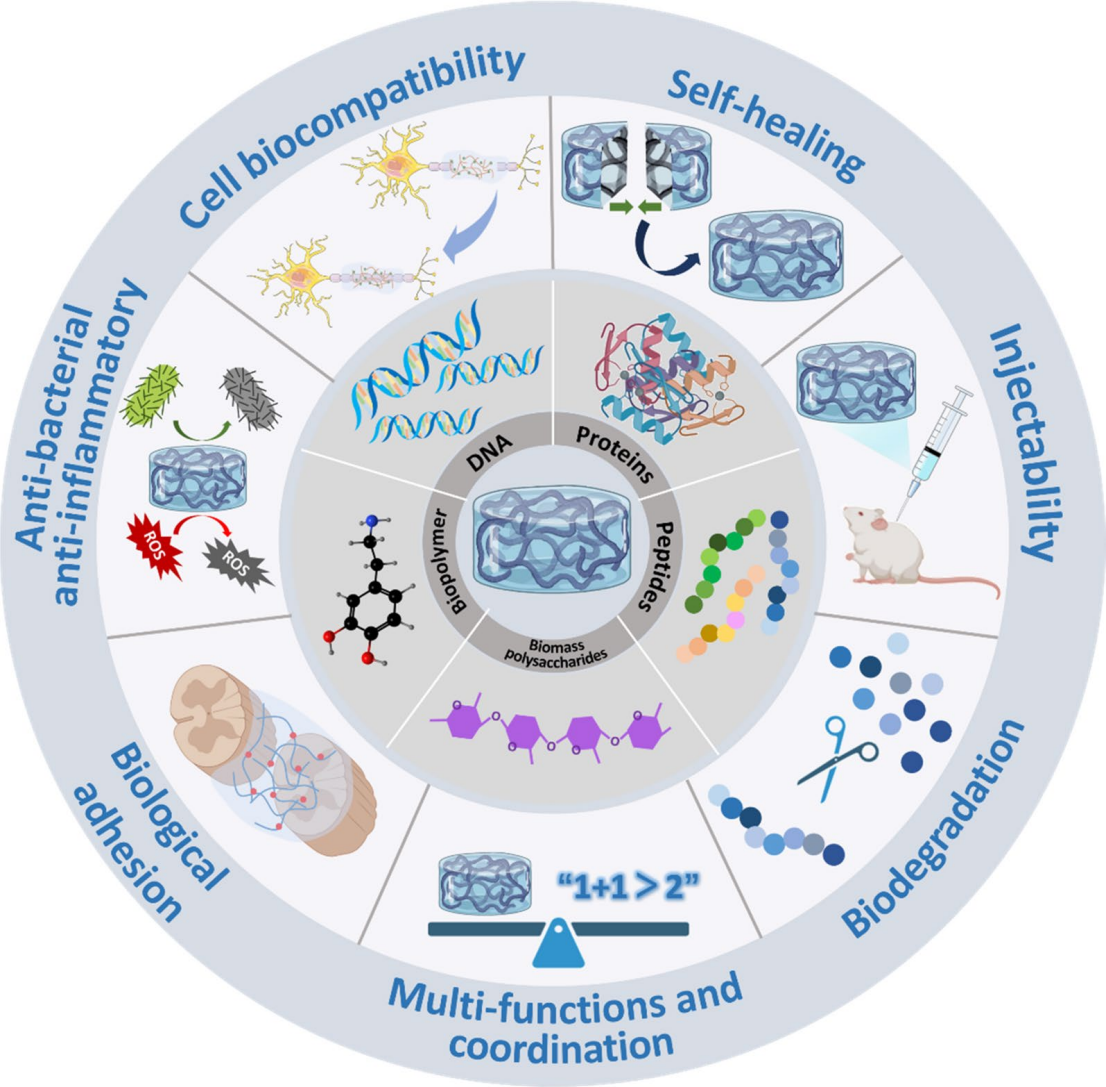
Model on the design and functional regulation of bioactive hydrogels for the SCI repair

Therefore, in this review we focus on recent advance in the material design and synthesis of functional bioactive hydrogels for repairing SCI, specifically, from the viewpoints of optimal material design and the regulation of the bioactivity and bio-functions of hydrogels (Scheme 1). Firstly, we introduce the SCI repair mechanisms and corresponding physical, chemical, and biological SCI repair methods. Secondly, we demonstrate the fabrication of bioactive hydrogels incorporating various biological components, including DNA, proteins, peptides, biomass polysaccharides, biopolymers, and others, via various synthesis strategies. After that, the methods for tailoring the biological properties of hydrogels, including cell biocompatibility, self-healing, anti-bacterial/anti-inflammatory, injection, bio-adhesion, biodegradation, and other multi-functions are presented. Finally, functional regulation of bioactive hydrogels through the functionalization of hydrogels with drugs/GFs, polymers, nanoparticles (NPs), one-dimensional (1D) materials, and two-dimensional (2D) materials for the SCI repair applications are introduced and discussed in detail, in order to show the great effects of functional regulation of hydrogels on treating SCI. We suggest, this comprehensive review analyze the importance of the functions and properties of bioactive hydrogels on the SCI repair, which could be useful for promoting the bridging between materials science and biomedicine in a different viewpoint and creating potential effects on clinical therapy of SCI.

### Mechanisms and methods of SCI repair

The spinal cord consists of both gray matter and white matter, with gray matter in the center and white matter in the periphery. Gray matter consists of interneuron, afferent neuron and efferent neuron fibers. White matter consists mainly of myelinated axons. The spinal cord provides a very efficient connection between the brain and peripheral nerves. Axons run lengthwise through the spinal cord, passing information from the brain to peripheral nerves via efferent nerves, and messages received by peripheral nerves to the brain via afferent nerves. Spinal cord neurons differentiate into axons and form synapses with dendrites, forming extensive and huge connections in the body. The effective connection of neurons can ensure the integrity and timeliness of information when the nervous system transmits signals.

Extensive progress has been made in the nerve regeneration of SCI. However, the existing studies still did not realize the regeneration of clinically meaningful regeneration of the adult CNS (i.e. restoration of motor, sensory, and autonomic nervous function), as it is not yet fully clear on the mechanisms for the recovery of the spinal cord function and the regeneration of the CNS. After reviewing the latest literature, several research mechanisms on SCI are summarized.

#### Mechanisms of SCI repair

Extensive progress has been made for the nerve regeneration of SCI. However, the existing studies still did not realize clinical regeneration of the adult CNS, as it is not yet fully clear on the mechanisms for the recovery of the spinal cord function and the regeneration of the CNS. SCI can be either primary or secondary, with the initial mechanical injury leading to a primary injury stage of the spinal cord that can last up to 24 h, resulting in the death of nerve and glial cells [22, 23]. Primary SCI is not treated clinically and can only be prevented, and the secondary SCI includes the breakdown of the blood-spinal barrier, the influx of peripheral inflammatory cells, and the activation of endogenous microglia, as well as other processes [24].

Secondary SCI can cause the activation of inflammatory cells, changes immune microenvironment, and further aggravate a series of pathophysiological events, such as neuron injury and glial cell population apoptosis, leading to the degeneration of ECM and the formation of cystic cavity and glial scar in the injured area eventually [25, 26]. Cystic cavities and glial scars impede electrical conduction of the spinal cord and the regeneration of axons, leading to severe dysfunction of the limbs below the injured level, such as permanent loss of movement (weakness or paralysis), sensory impairment, and autonomic nerve (defecation and urination) dysfunction [27, 28]. The neurons are divided into the axons and form synapses with dendritic nodes, which form a wide and large connections in the body, which can ensure the integrity and functions in the signaling system. However, the regeneration ability of the axon and dendrites is often inhibited by a large degree of inhibition, including the loss of the nerve functions and their effects of the inhibitory microenvironment (glia scar formation, inflammatory stimulation, and oxidative stress) [29].

There are many other studies on exploring the mechanisms of the SCI repair. For instance, it has been reported that the mammalian target protein of rapamycin (mTOR) signaling pathway played an crucial role in the synaptogenesis, neuron growth, differentiation, and survival after the injury of CNS [30]. The modulation of mTOR signaling pathway is a potential treatment for SCI. After SCI, the astrocytes have become hypertrophic and prolifically, forming borders rich in astrocytes, and then overreact to form glial scars, which are the main obstacles to neuronal regeneration and axon recovery[31]. Previously, it has been reported that the down-regulated PI3K/Akt/mTOR signaling pathway reduced the formation of glial scars, promoted the autophagy of neuronal cells after SCI, inhibited the apoptosis, and improved functional recovery in rats of SCI [32–34]. Several studies have proved that the activation of the PI3K/Akt/mTOR pathway was beneficial to the SCI repair. For example, Sun and co-workers reported that the combination of bone marrow mesenchymal stem cells (BMSCs) with exercise therapy restored the motor function after SCI by activating the PI3K/Akt/mTOR pathway [35]. Zhan and co-workers found that moderate intensity treadmill exercise activated the mTOR pathway, which was dependent on the expression of neurotrophic factors in the motor cortex, and promoted functional recovery in mice of SCI [36]. In addition, previous studies [37, 38] have also suggested that ATP could promote functional recovery of SCI rats by activating the mTOR signaling pathway. Therefore, the mTOR signaling pathway mechanism plays an important clinical role in the formation of glial scar, the survival, proliferation, and differentiation of NSCs, as well as the growth, differentiation, and survival of neurons after SCI.

Both glial scar and scar mechanism, which are formed mainly by reactive astrocytes, play a dual role in SCI [39]. In the acute stage of SCI, the astrocytes will secrete various GFs to renew their numbers, which not only have direct effects on the damaged nerve cells, but also reduce the concentration of toxic substances in the external environment glutamate. These efforts removed harmful substances from the extracellular fluid, and mobilized energy to the injured area, so that the living environment of nerve cells was repaired [40, 41]. However, in chronic phase, hypertrophic glial scars formed by reactive astrocytes have physical and chemical barriers, which are the key culprit of hindering neuron regeneration and functional recovery [42, 43]. The complexity of reactive glial scar formation in spinal axon regeneration and functional recovery has been discovered previously [44]. The obtained results indicated that there was no significant difference in the recovery of animals with and without glial scar resection in a dorsal semi-resection model of experimental animals. However, the blood–brain barrier (BBB) score of the contusion model animals was lower in the early postoperative glial scar resection group, which confirmed the duality and complexity of glial cell response after SCI.

Besides, emerging research is elucidating the mechanism of neural circuit recombination after SCI to improve the functional recovery of SCI. Researchers are trying to understand how the subsets of neurons from the brain stem and spinal cord interact to regulate the motor and autonomic functions. Their study also explained the response and recombination of these subsets of neurons after SCI, and presented an effective strategy to improve the function of SCI through the neuromodulation technique [45].

#### Methods of SCI repair

The current treatment strategies for the SCI include the protection of the nerve cells and the regeneration of the nerve cells [46]. The former strategy is mainly used to avoid secondary SCI and plays a positive role in the early stage of SCI. There are two common therapeutic measures for acute SCI. One is releasing the continuous mechanical compression of the spinal cord, such as early surgical spinal decompression and spinal fixation, and the other is reducing acute inflammatory reactions [23]. For example, high-dose methylprednisolone has been used to treat acute SCI within 48 h after the injury, but its side effects were serious and the treating performance was limited [31]. Other strategies have been developed to repair and regenerate nerve tissue and restore its function. For example, the transplantation of stem cells and the stimulation of the proliferation and differentiation of endogenous NSCs for the SCI repair have been reported, and clinical achievements have been obtained for protecting and repairing the damage of CNS [27, 47]. Transplanted stem cells or activated endogenous NSCs are helpful to repair the damaged spinal cord nerve cells and play important role in promoting SCI repair through immune regulation or cell regeneration. However, the success rate of stem cell transplantation in the clinical stage is very low, mainly due to the poor viability of cells and poor integration of spinal cord tissue [48].

The successful clinical method for the treatment of chronic SCI patients is the bionic epidural electrical stimulation (EES). For instance, Andreas and co-workers have used the bionic EES to restore three patients with chronic paralysis to standing, walking, cycling, swimming, and torso control within one day [43] Two of the participants were able to regulate the movement of the leg during the treatment of the EES, indicating that the stimulus increased the signal of the remaining down path. The bionic EES also achieved positive and continuous motion in the early stages of SCI, and made full use of natural repair mechanisms to enhance the recovery of the nervous system. This technique opens a practical avenue by applying clinical therapies for effective treatment of patients with severe SCI.

#### Hydrogel materials for SCI repair

The spinal cord is a soft watery biological structure with stiffness that can range from 3 to 300 kPa. As a kind of biological nanomaterial, hydrogel has unique advantages for repairing SCI due to its high hydrophilicity and other physical properties. Previous study has indicated that the maturity of neurons was higher and the length of axon was increased after using hydrogels, which was more suitable for the implantation after SCI and conducive to the regeneration of spinal cord tissue [49].

**Fig. 1:**
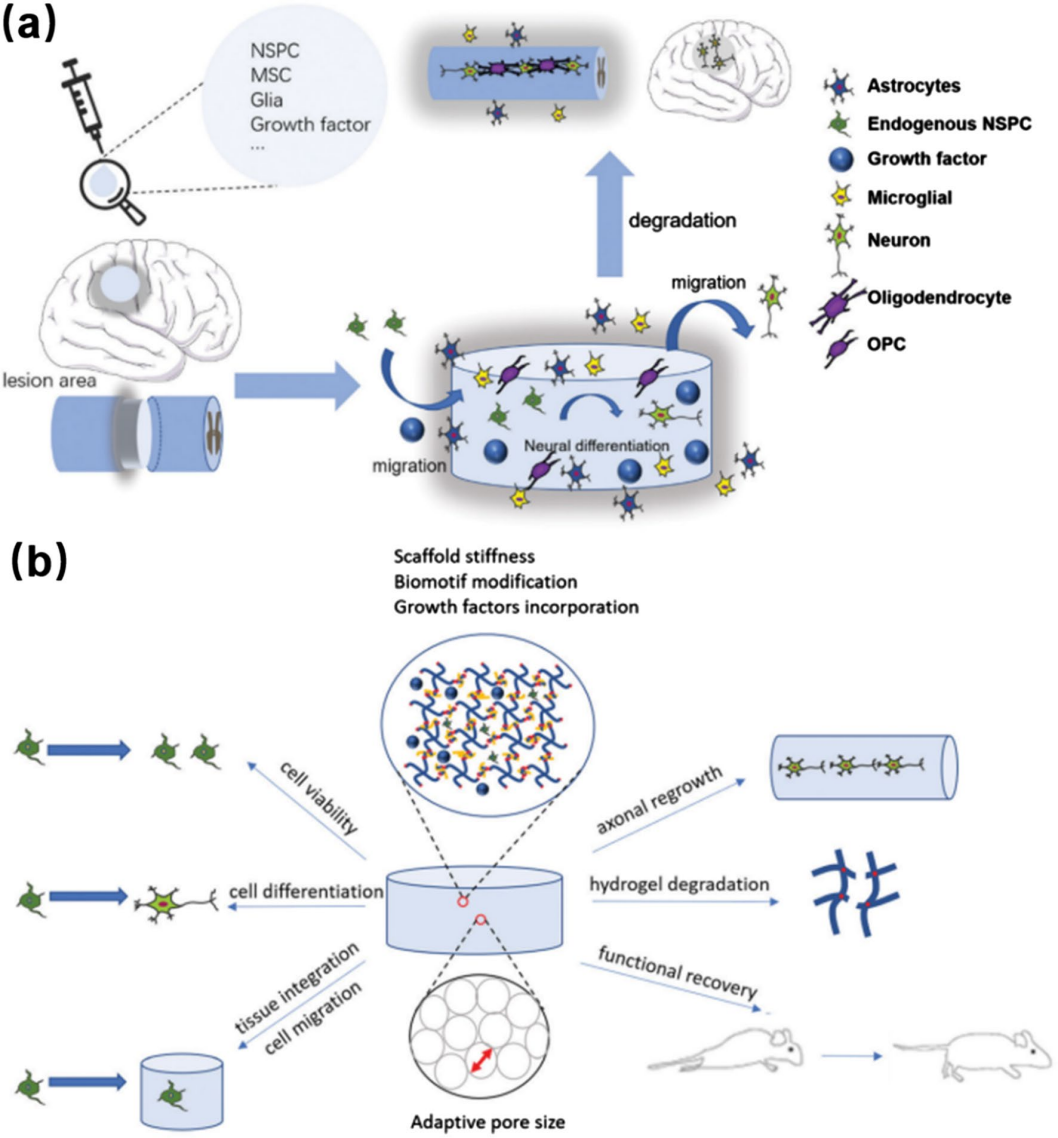
Hydrogels for tissue engineering applications: **a** Diagram of hydrogels treatment of central neuropathy (brain, spinal cord). **b** Cell behavior of injectable hydrogels. Reprinted from Ref [50], Copyright 2021, Royal Society of Chemistry

Hydrogels are highly hydrating materials with water molecules and hydrophilic polymer networks. Their injectability, inherent biocompatibility, cell interaction, hydrophilicity, permeability, and biodegradability make them suitable substrates for simulating natural molecular microenvironments. As shown in Fig. 1a, b a recent review has indicated that injectable hydrogels could be used for the stem cell transfer, and the selection of hydrogel materials will be mainly based on the spatial structure, as well as the tissue and cell reactions with nanomaterials [50].

Hydrogels can not only be used as ideal scaffolds for nerve tissue engineering, but also provide biological microenvironments for electrical stimulation [51]. The injection of hydrogels into the injured sites of SCI has been proved to be a facile way for drug delivery and the repair of SCI. In the case of SCI, the injectable nature of hydrogels provides a clinical advantage compared to other traditional treatments, which is especially suitable for clinical minimally invasive surgery of SCI therapy [52]. The specific gel that simulates the CNS microenvironment has been utilized to improve the transplantation of exogenous stem cells and activate the survive of endogenous NSCs [53]. With good biocompatibility, hydrogels can form scaffolds *in-situ* to fill the irregular shape of the defect tissue, eliminate the space after SCI, guide stem cell infiltration and matrix deposition, and create a complete implant-tissue interface to restore the continuity of the SCI tissue and achieve the SCI repair [54, 55].

**Fig. 2:**
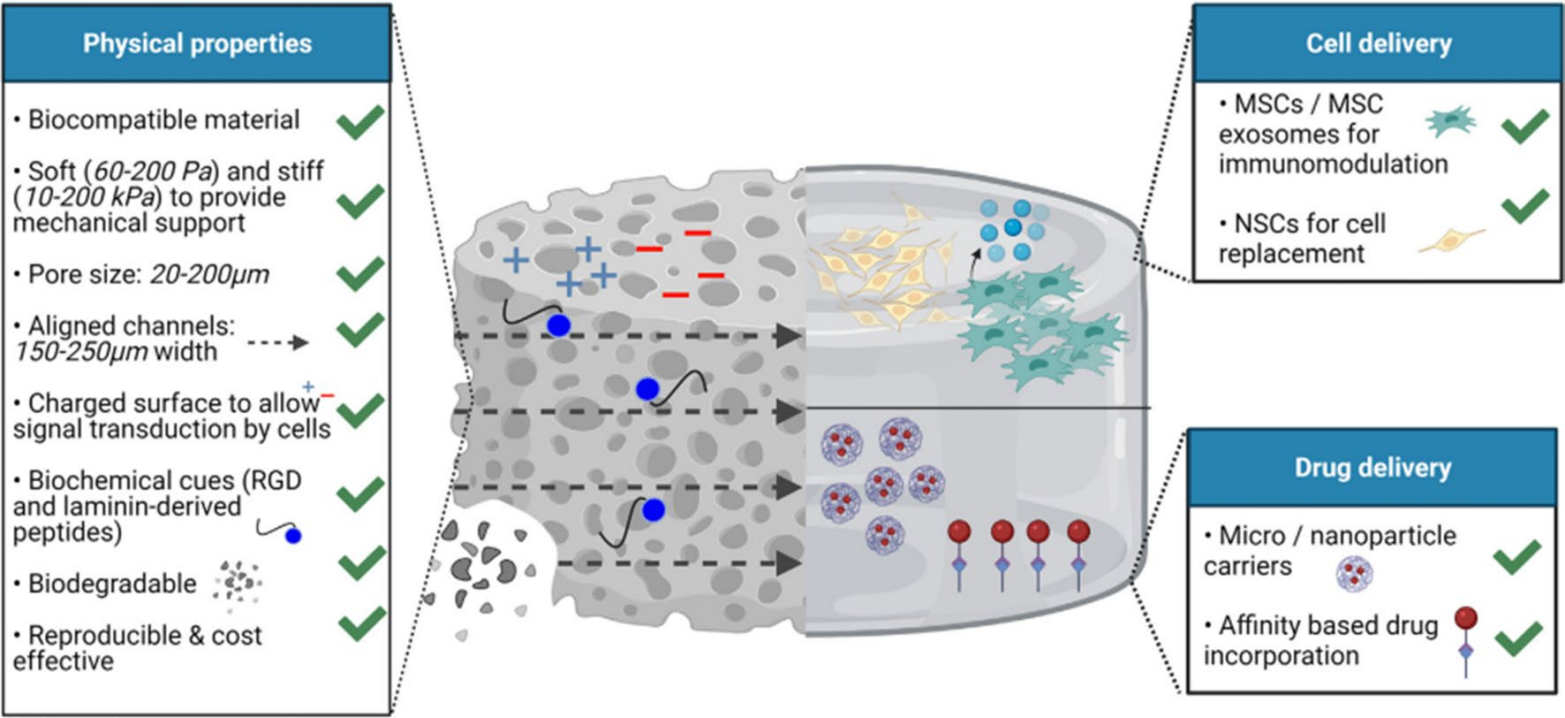
Unique physical, chemical, and biological properties of hydrogels for cell and drug delivery in SCI repairing. Reprinted from Ref [14], Copyright 2022, Elsevier

Hydrogels with unique physical, chemical, and biological properties can be used for repairing SCI through loading cells and drugs to the injured sites [14]. As shown in Fig. 2, porous and aligned structured hydrogels with high biocompatibility and biodegradation can support molecular mobility and the regeneration of linear axon within hydrogels for the SCI repair. In addition, the adjustable mechanical properties and minimally invasive delivery of cells and drugs make them more attractive carries for pharmaceutic treating of SCI, by which cells, drugs, and GFs can be loaded into hydrogels and then released into the SCI systems. Compared to traditional drug delivery carriers, the using of hydrogels as drug carriers can promote sustainable release of drugs or GFs and avoid the blood-spinal barrier [56, 57]. Besides, due to the doping of active GFs/drugs into a cross-linked hydrogel matrix via electrostatic interactions or chemical binding, the formed bioactive hydrogels exhibited better protection from enzymatic biodegradation and rapid de-activation [58].

Although hydrogel has many properties suitable for the repair of spinal cord injury, it can have some defects. Low mechanical stability, high cost, variability, and poor immunogenicity are still an obstacle to the application of hydrogel in SCI [59]. Therefore, the development of hydrogels with more excellent properties, and continuous optimization of the biomedical application of hydrogels are important links in the application of broadened hydrogels in the repair of spinal cord injury [60].

### Fabrication of bioactive hydrogels

Bioactive hydrogels can be synthesized by the cross-linking various biological components or modifying the polymer hydrogels with various biomolecules. In this section, the strategies for fabricating bioactive hydrogels using DNA, proteins, peptides, biomass polysaccharides, biopolymers, and others are introduced.

#### DNA hydrogels

DNA hydrogels have become a type of widely studied bioactive nanomaterials in biomedicine ascribing to their high biocompatibility, controllable properties, packaging, and delivery ability [61]. For example, DNA hydrogels have shown excellent performance in drug/gene delivery, bone tissue engineering, and healthcare sensors. In particularly, DNA hydrogels have been proved to be effective drug delivery platforms as they can encapsulate and release drugs in a continuous and controlled manner [62].

**Fig. 3:**
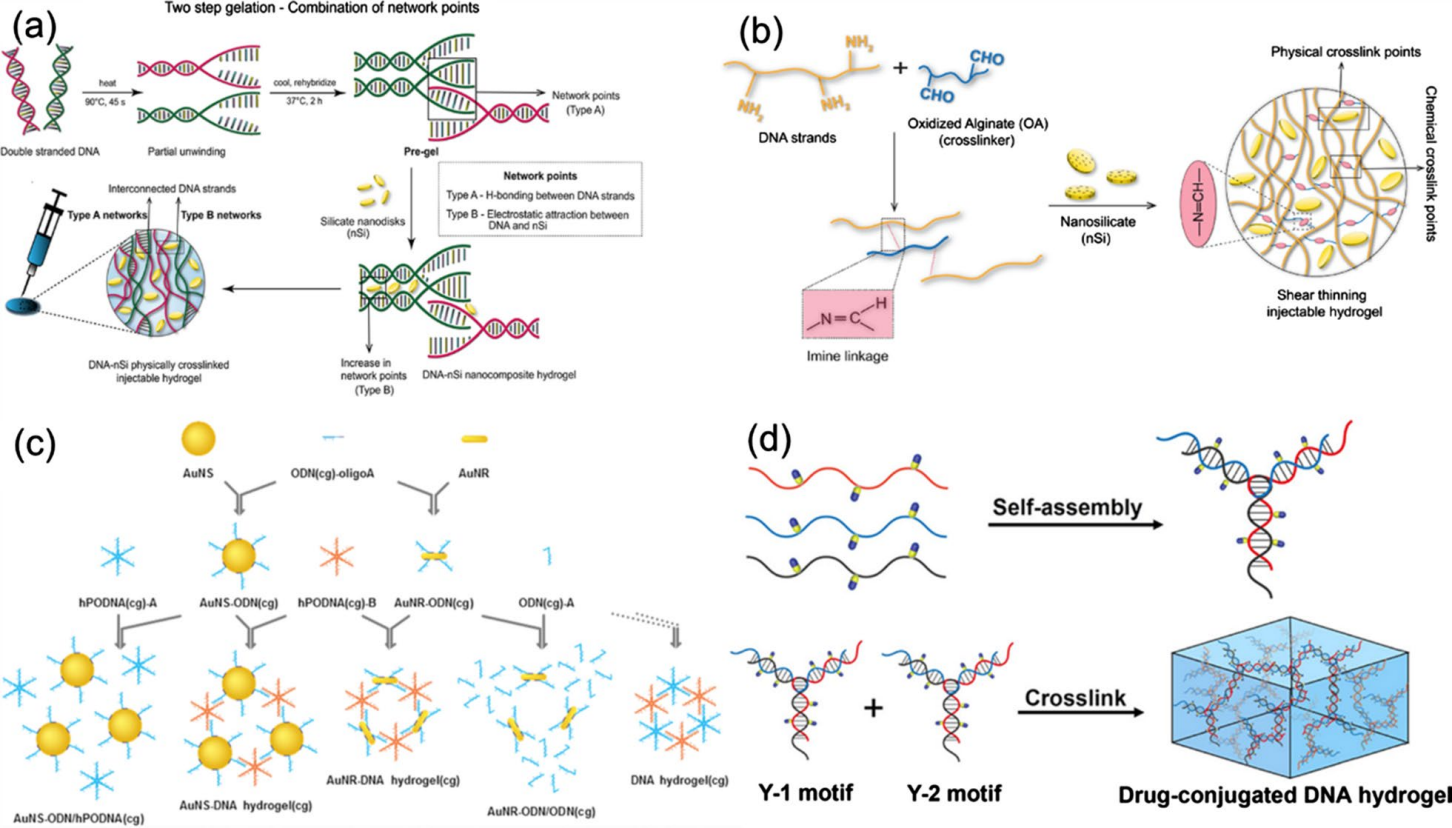
The preparation process and structure diagram of bioactive DNA hydrogels: **a** DNA-nSi hydrogels. Reprinted from Ref. [63], Copyright 2018, American Chemical Society. **b** DNA-OA-nSi hydrogels. Reprinted from Ref. [64], Copyright 2020, Elsevier. **c** AuNS-DNA and AuNR-DNA hydrogels. Reprinted from Ref. [65], Copyright 2017, Elsevier. **d** CPT-DNA hydrogels. Reprinted from Ref. [66], Copyright 2020, American Chemical Society

Basu and co-workers reported the preparation of DNA-nSi nanocomposite hydrogels for the applications in tissue engineering and drug delivery. The DNA-nSi hydrogels were prepared using simple heating and mixing techniques through a physical cross-linking network that formed between DNA and silicate nanodisks (nSi) [63]. As shown in Fig. 3a, the gelation process consists of two steps. In the first step, DNA denaturation and re-hybridization were used to form the hydrogen bonds between complementary base pairs of adjacent DNA chains. Secondly, nSi were used to create additional network through attractive electrostatic interactions with the DNA trunk, thereby enhancing mechanical elasticity of the created DNA hydrogels. The thermal stability and mechanical properties of the formed DNA hydrogels could be adjusted by changing the concentration of nSi. The hydrogel exhibited good biocompatibility and sustained drug release properties. It is proved that the hydrogels could regulate the release of the model drug dexamethasone (Dex). In the rat skull defect model, the DNA-nSi hydrogels have been testified to be effective to enhance the osteogenic differentiation and bone formation of human adipose stem cells. This study presents a new method for the preparation of injectable hydrogels and provides a new choice for the applications of hydrogels in tissue engineering, medical device coating, and drug delivery.

Injectable self-healing hydrogels have been introduced in another similar study, in which the hydrogels were fabricated using the components of DNA, oxidized alginate (OA), and nSi [64]. As shown in Fig. 3b, DNA-OA chains are connected using the Schiff base reaction between the aldehyde group of OA and the amino group of DNA nucleotides to form a covalent bond. The reversibility of the cross-linking reaction provided shear-thinning and self-healing properties for the formed DNA-OA network structure. In addition, the addition of nSi induced the formation of additional physical cross-linking sites, thus enhancing mechanical strength of DNA hydrogels without affecting their self-healing properties and biocompatibility. The fabricated DNA-OA-nSi hydrogels acted as injectable carriers for continuous delivery of the hydrophobic drug with a half-life of about 5 days and showed no any cytotoxicity. The obtained results confirmed the bioactivity of the released drugs by testing their ability to induce osteogenic differentiation in vitro and the migration of human adipose-derived stem cells. In addition, the designed DNA-based hydrogels could be used for continuous delivery of small molecular drugs that similar to simvastatin, showing their wide applications.

In addition, some DNA molecules with special functions can also be designed and prepared into hydrogels. For instance, Yata et al. designed a compound immunostimulatory DNA hydrogel, which consisted of a mixture of specific DNA sequences containing cytosine (C) and guanine (G) that separated by the phosphate groups (CpG) and gold nanospheres (AuNS) modified with DNA (hPODNA) [65]. As shown in Fig. 3c, ODN-modified AuNS was firstly synthesized and named as AuNS-ODN (cg) and AuNS-ODN (gc), by adsorbing CpG or GpC with oligodeoxynucleotides (ODN) onto the surface of AuNS. Then, AuNS-ODN (cg) and hPODNA (cg) were mixed to form the AuNS-DNA composite hydrogels. In the experiment, EG7-OVA tumor-bearing mice were treated with the formed AuNS-DNA hydrogels under the irradiation of 780 nm laser, which significantly inhibited the growth of tumor cells and prolonged the survival time of mice. The composite hydrogels had high biocompatibility and safety, and could be removed from the blood by mononuclear phagocytic system. After laser irradiation, the hydrogels released DNA and stimulated immune cells to release proinflammatory cytokines and induced strong anti-tumor immune response.

In another study, Zhang et al. designed an injectable DNA hydrogel with chemotherapy function to solve the problem of tumor recurrence [66]. As shown in Fig. 3d, camptothecin (CPT) was transplanted into the backbone of thiophosphate DNA to form DNA-drug conjugate (DDC) chains, which were then assembled into Y-shaped drug-loaded DNA hydrogels. Compared with traditional systemic chemotherapy, this drug-containing DNA hydrogel exhibited a sustainable and responsive drug release behavior, which significantly inhibited the regeneration of tumor cells and prevented tumor recurrence [66]. Meanwhile, its local administration of minimally invasive treatment can also avoid organ damage that caused by the toxicity of systemic chemotherapy. The designed hydrogel showed a continuous and responsive drug release behavior, which could well infiltrate into the residual tumor tissue and be absorbed by cells effectively. The design and preparation of this drug-containing DNA hydrogel provide a promising solution for local adjuvant therapy of tumor.

#### Protein hydrogels

Various protein hydrogels shows good mechanical properties and high biocompatibility, both of which can be finely regulated by adjusting the synthesis conditions of hydrogels [67, 68]. The preparation of protein hydrogels is simple and feasible, which provide functional biomaterials for the tissue regeneration and therapy of stem cells. In addition, protein hydrogels are injectable and self-healing, which make them more promising for various applications [69]. At present, a variety of proteins can be used as raw materials for the preparation of hydrogels, such as silk fibroin, zein, gelatin, elastin and keratin [70, 71]. This section mainly introduces some hydrogels prepared by silk fibroin and its derivatives, as well as some protein hydrogels with special functions.

For example, Wang et al*.* reported in their study a method for introducing inert silk fibroin nanofibers (SFN) to form SF hydrogels in an enzymatic crosslinking system for regenerating silk fibroin (RSF) [72]. The mechanical properties of the formed SF hydrogel were tunable and could guide the differentiation behavior of stem cells. During the preparation process, RSF formed dityrosine bonds in the presence of horseradish peroxidase (HRP) and then cross-linked to form a hydrogel, in which SFN was embedded in the RSF hydrogel matrix to improve its mechanical properties. By adjusting the amount of added SFN, the stiffness of the SF hydrogel was regulated to about 9–60 kPa, which was much higher than that of hydrogel without SFN (about 1 kPa).

**Fig. 4:**
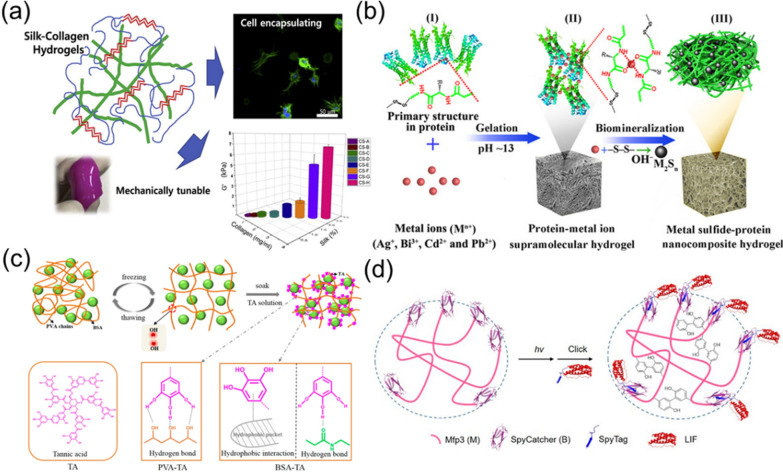
Synthesis and structures of bioactive protein hydrogels: **a** SF-collagen composite hydrogels. Reprinted from Ref. [73], Copyright 2017, Elsevier **b** Metal sulfide-protein hybrid hydrogels. Reprinted from Ref. [75], Copyright 2017, Wiley–VCH. **c** TA-PVA/BSA hydrogels. Reprinted from Ref. [76], Copyright 2018, American Chemical Society. **d** Mfp3 hydrogels formed by photochemical gelation. Reprinted from Ref. [78], Copyright 2018, American Chemical Society

Protein hydrogels prepared by combining SF as the main component with other bioactive materials exhibited enhanced biological functions. The Buitrago team studied a hybrid protein hydrogel composed of SF and collagen, which showed improved flexibility and tunability that individual protein materials did not have (Fig. 4a) [73]. The mechanical and biological properties of the formed hydrogel were tailored by adjusting the ratio and concentration of SF and collagen, and the stiffness ranged from 0.017 to 6.81 kPa. The biological test with cells indicated that the hydrogel promoted the cell growth, differentiation, and muscle cell formation. Besides, the hydrogel regulated the synthesis and distribution of ECM, thereby better promoted the cell regeneration and tissue repair. In a previous study, Raia and co-workers reported the development of composite hydrogels of SF and hyaluronic acid (HA) for tissue engineering application [74]. SF and HA were covalently cross-linked under enzymatic reaction to form composite hydrogels, which revealed tunable mechanical properties and degradation ability. By adjusting the concentrations of SF and HA, the formed hydrogels exhibited a wide range of stiffness, from 10 kPa to slightly below 1 MPa. In addition, the designed SF-HA hydrogels revealed promising degradation ability, cytocompatibility, and elasticity, making the hydrogels good candidates for long-term tissue engineering applications.

In addition to SF, other proteins with special functions can also be constructed into bioactive hydrogels. Wang et al*.* proposed a method to construct composite hydrogels with injectable and self-healing properties through the formation of dynamic protein-metal ion network [75]. As shown in Fig. 4b, metal ions were mixed with protein under alkaline conditions to form a complex network under the interactions between metal ions and the cysteine residues of proteins. Nanocomposite hydrogels were synthesized by the *in-situ* reduction of metal ions into small-sized metal sulfide NPs. In the experiment, Bi^3+^ was added into bovine serum albumin (BSA) to form the Bi_2_S_3_-BSA hydrogel for photothermal therapy of tumors. The Bi_2_S_3_-BSA hydrogel exhibited injectable and self-healing properties, as well as high photothermal efficiency. The designed injectable, self-healing, and adaptable hydrogel showed several biomedical applications, especially in tissue regeneration and stem cell therapy.

In another case, BSA protein was also used to build high-strength protein hydrogels through non-covalent interactions [76]. As shown in Fig. 4c, tannic acid (TA), BSA, and polyvinyl alcohol (PVA) were mixed together to form TA-PVA/BSA hydrogel via physical cross-linking. The pre-hydrogel was prepared from BSA and PVA by repeated freezing and thawing, which was then soaked in TA solution to form cross-linked TA-PVA/BSA hydrogel. Compared with traditional hydrogels, the TA-PVA/BSA hydrogel revealed ultrahigh tensile strength up to 9.5 MPa, and had good water-retention and similar layered structure to human skin. Furthermore, the hydrogel possessed tunable mechanical properties and anisotropy. These unique properties promoted the biological applications of designed protein hydrogels.

When stimulated by external or internal factors, such as metabolic product concentration, pH value, light/UV source, enzymes, osmotic pressure, magnetic/electric field, temperature, redox reactions, and ultrasound irradiation, stimulus-responsive hydrogels exhibit significant changes in their swelling, degradation, rheological properties, release behavior, and mechanical performance. Therefore, by achieving and controlling these stimulus conditions, researchers are able to fabricate stimulus-responsive hydrogels with adjustable properties. Additionally, the use of protein precursors with stimulus-responsive functionality can also confer stimulus-responsive properties to hydrogels [77]. In a typical case, Liu et al*.* [78] presented the design of a protein hydrogel by photochemical cross-linking of recombinant mussel foot protein-3 (Mfp3), as shown in Fig. 4d. The mechanical properties of the designed protein hydrogel could be regulated by adjusting the protein concentration, the co-oxidant concentration, and the intensity of light used for cross-linking during the preparation process. The protein hydrogel had good biocompatibility to support cell adhesion and proliferation, and could modify and immobilize leukemia inhibitory factor under covalent interaction to activate the JAK/STAT3 pathway to induce neuronal growth. The material design with folded protein domains and photochemical gelation was beneficial to construct bioactive materials for regenerative neurobiology [78].

#### Peptide hydrogels

Peptide hydrogels showed high potential for biomedicine, which were excellent bioactive materials for the wound repair, cell culture, and drug/gene delivery [79]. In order to achieve better remote and precise control of hydrogel properties, researchers have proposed different strategies, including the using peptides with special bioactive functions to construct multifunctional hydrogels, using photo-sensitive peptides to construct hydrogels, and using self-assembled biomimetic hydrogels [80].

**Fig. 5:**
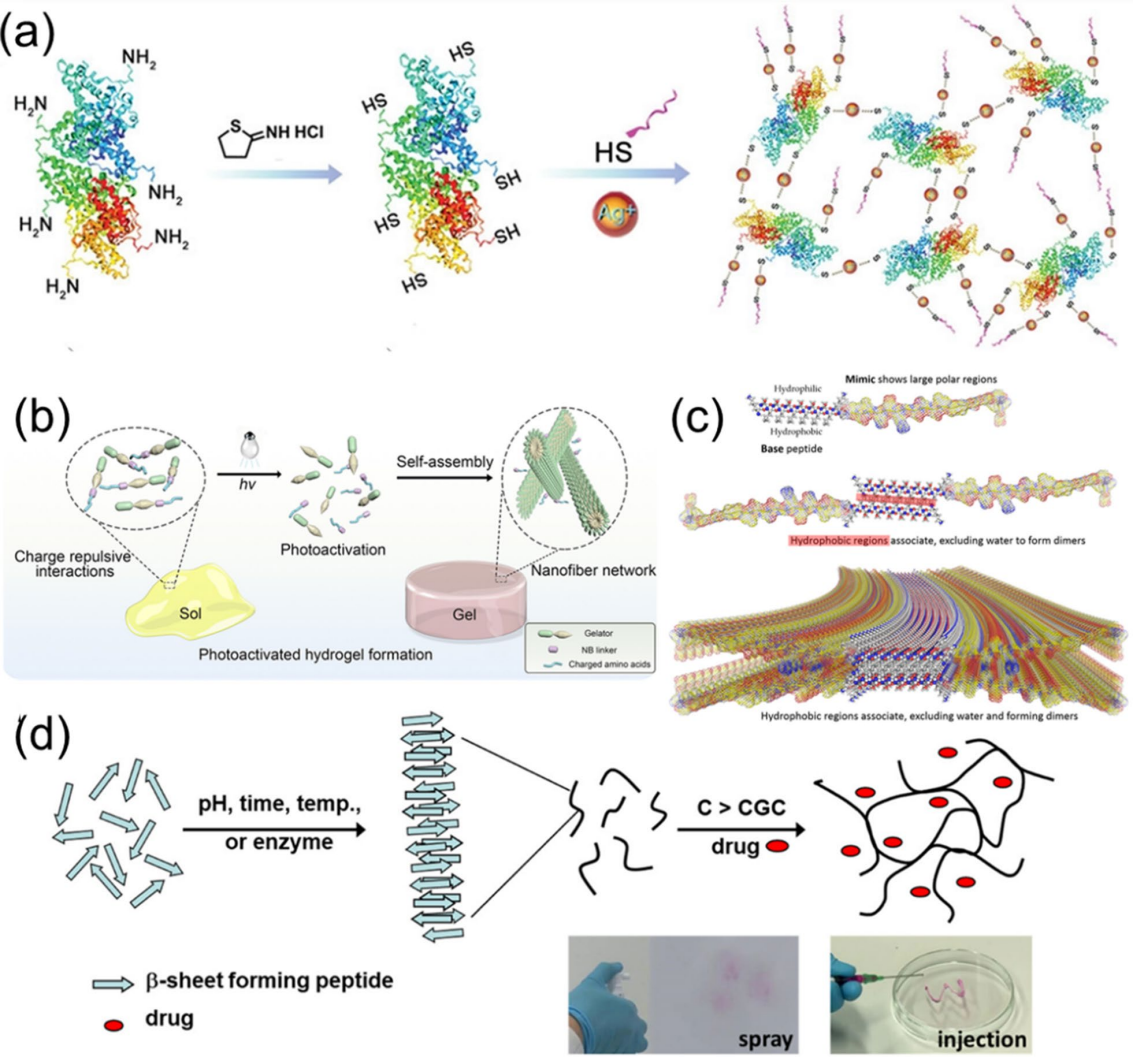
Synthesis and structures of bioactive peptide hydrogels: **a** KK-BSA hydrogels formed by Ag–S coordination. Reprinted from Ref. [81], Copyright 2020, Wiley–VCH. **b** Photosensitive peptide hydrogel via self-assembly. Reprinted from Ref. [82], Copyright·2023, American Chemical Society. **c** ECM protein-mimic peptide hydrogel. Reprinted from Ref. [83], Copyright 2018, American Chemical Society. **d** Self-assembly and gelation pathways of β-sheet forming peptides. Reprinted from Ref [84], Copyright 2022, Royal Society of Chemistry

For instance, Cheng et al*.* introduced a new type of polypeptide-protein hydrogel that formed by cross-linking BSA, K_2_(SL)_6_K_2_ polypeptide (KK), and (Ag^+^) [81]. The hydrogel was formed by the S–Ag coordination and the cross-linking of BSA protein, thiol polypeptide K_2_(SL)_6_K_2_ polypeptide (KK), and Ag^+^ (Fig. 5a). The formed KK-BSA hydrogel revealed good gel effect, rich porous structure, and self-healing property. In terms of targeting wound healing, Ag^+^ provided antibacterial function, and KK endowed the hydrogel with the property of promoting blood vessel growth. The in vivo experiments in mice indicated that the KK-BSA hydrogel promoted considerable collagen deposition and vascularization capacity in the early stage of wound healing, favoring the generation of newly emerging hair follicles. This peptide-protein hybrid hydrogel with antibacterial and vascularizing properties helped to regenerate and heal infected wounds through synergistic effects of a few components.

The self-assembly of photoactivate peptide is a general approach to construct peptide hydrogels with spatial and temporal control. In a recent report, Xiang et al. proposed a new strategy of using photosensitive peptides to construct bioactive hydrogels, which were triggered under the light irradiation to achieve remote and precise control of hydrogel properties. This strategy involved designing peptide molecules with high aggregation ability, charged amino acid sequences for preventing the self-assembly in water, and photocleavable linkers to activate peptide self-assembly upon the light irradiation [82]. As shown in Fig. 5b, a photo-responsive peptide modified with the gelling agent, a charged amino acid sequence, and a 2-nitrobenzyl (NB) ester photocleavage group was designed to activate the peptide self-assembly under the light irradiation. The designed peptide formed bioactive hydrogels in neutral aqueous solutions under the UV irradiation, which opened up the possibility of mimicking ECM and showed potential applications in cell culture and tissue engineering.

Self-assembled peptide hydrogels are useful for drug delivery. Nguyen et al. used self-assembling peptides to prepare biomimetic hydrogels, which promoted the regeneration of dental pulp stem cells [83]. As shown in Fig. 5c, the self-assembling peptide mainly contains a β-sheet-forming segment and an ECM phosphoglycoprotein-mimic sequence at the C-terminus. The presence of hydrophilic and hydrophobic residues enabled the peptide to self-assemble into β-sheet stacking nanofibers. Biodegradable and injectable properties of the formed peptide hydrogels could be tailored by adjusting the solution pH. Meanwhile, the fabricated hydrogels revealed rheological properties, making them easy to be injected into the injured sites to promote the survival and proliferation of autologous stem cells and the formation of dental bone.

In another work, Elsawy and co-workers introduced the potential application of self-assembled peptide hydrogels for drug delivery using five β-sheet peptides (F8, FK, FE, F8K, and KF8K) with different physicochemical properties [84]. As shown in Fig. 5d**,** the self-assembly pathways and the doping of drugs (Dox) into the hydrogels are presented. Their results indicated that the ion-π and π-π interactions between drugs and peptide nanofibers affected the release of Dox. In addition, the created peptide hydrogels exhibited broad susceptibility to enzymatic degradation, which could be exploited to control the degradation rate. In addition, the Dox released from the hydrogels was pharmaceutically active and could affect the cell growth. Their study demonstrates the potential of self-assembled peptide hydrogels as a platform for drug delivery.

#### Biomass polysaccharide hydrogels

Biomass polysaccharides can also be used to construct hydrogel materials with a wide variety and diverse structures, which have attracted great attention in the fields of drug delivery and wound repair [85, 86]. In the past few years, various types of polysaccharide hydrogels have been prepared through different methods, and their properties and applications in various fields have been explored. This section introduces the preparation method, physicochemical properties, bioactivity, and applications of polysaccharide hydrogels.

**Fig. 6:**
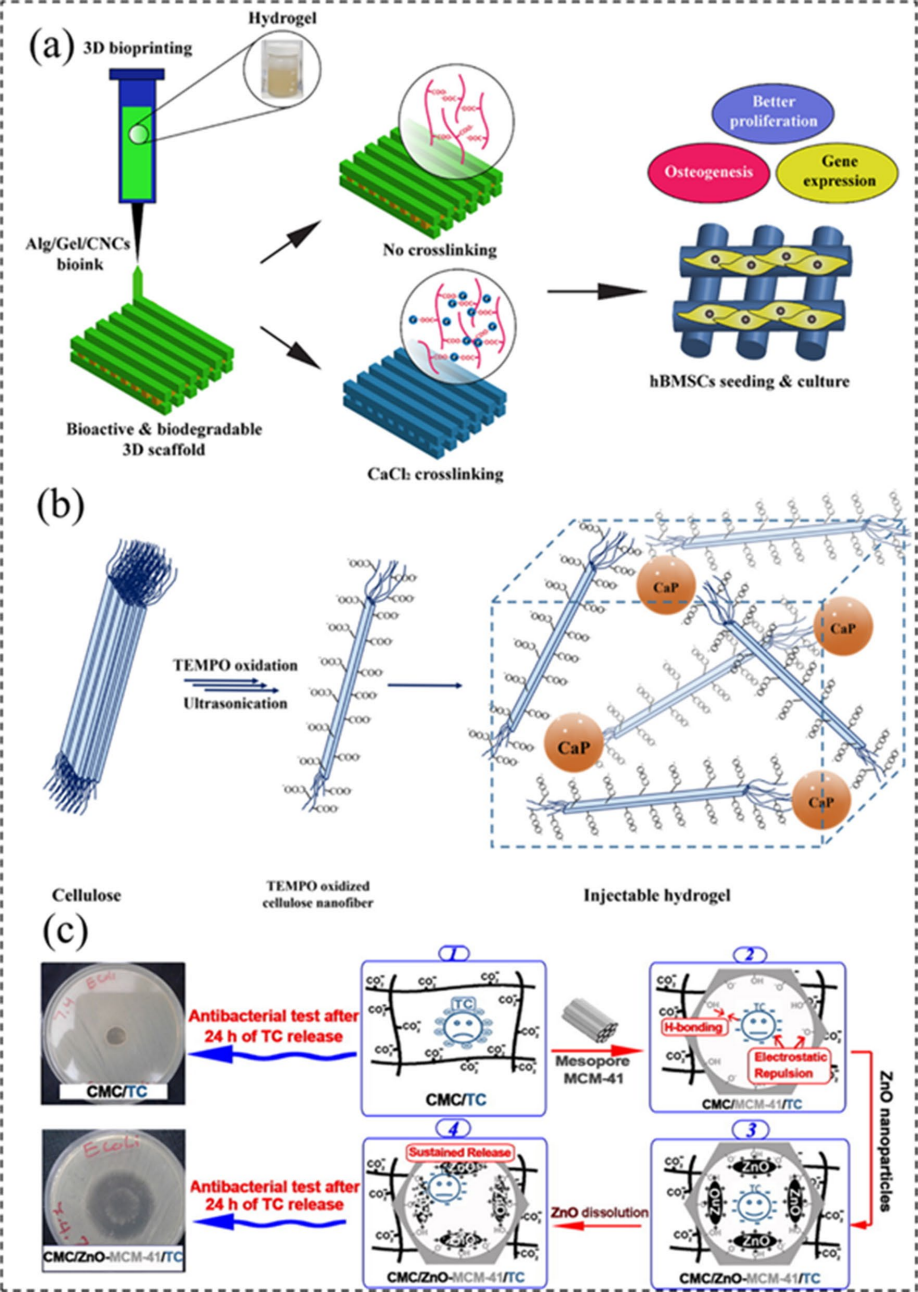
Synthesis and structure of bioactive polysaccharide hydrogels: **a** 3D printed Alg/Gel/CNCs hydrogel. Reprinted from Ref. [87], Copyright· 2021, Elsevier. **b** CaP-TOCNF hybrid hydrogel. Reprinted from Ref. [88], Copyright·2021, MDPI. **c** CM/ZnO-MCM-41/TC hybrid hydrogel for drug delivery. Reprinted from Ref. [90], Copyright 2017, Elsevier

Dutta et al. utilized 3D printing technology to fabricate a biodegradable hybrid hydrogel for bone tissue engineering by using alginate (Alg), gelatin (Gel), and cellulose nanocrystals (CNC), as shown in Fig. 6a [87]. In their experiment, the Alg/Gel/CNC hydrogel-based bioink was prepared by physical and Ca^2+^-induced chemical cross-linking, which showed enhanced mechanical properties compared with pure polymer scaffolds. The biocompatibility, cell differentiation, and bone regeneration ability of the printed scaffolds were evaluated using various assays, and the results showed that the 1% Alg/Gel/CNC hydrogel scaffolds revealed enhanced cell adhesion and proliferation, as well as mineralization and osteogenesis compared to the control group. Their study provides a new approach to develop bioactive hydrogel materials for tissue engineering.

In another work, Fiorati et al. regulated the mechanical properties of 2,2,6,6-Tetramethyl-1-Piperidinyloxy (TEMPO)-oxidized cellulose nanofibers (TOCNFs) by adding inorganic nanoparticles, while keeping the injectability and bioactivity of the cellulose hydrogel (Fig. 6b) [88]. In their study, calcium phosphate (CaP) NPs were embedded into the injectable TOCNF hydrogel for inducing the mineralization to form hydroxyapatite layers for bone tissue regeneration. The formed CaP-TOCNF hybrid hydrogel exhibited good stability, high injectability and biological activity, as well as excellent biocompatibility, providing valuable insights on the design and synthesis of natural polymer-based hydrogels for tissue engineering applications.

Shah and co-workers developed the synthesis of an injectable hydrogel from chitosan (CTS), carboxymethylcellulose (CMC), and PF127 (Pluronic^®^ F127) using the solvent casting technique, which was further loaded with curcumin (Cur) to promote the diabetic wound healing [89]. The fabricated injectable CTS-CMC-g-PF127 hydrogel exhibited good mechanical properties, rheological properties, and thermal responsiveness. In addition, the biotests indicated that the created hybrid biomass hydrogel revealed better ability for diabetic wound healing by promoting the tissue regeneration, inhibiting the inflammatory cells, and increasing the angiogenesis. In a similar case, Rakhshaei and co-workers used citric acid as a cross-linking agent to fabricate a flexible nanocomposite hydrogel of CMC, ZnO-modified mesoporous silica (MCM-41), and tetracycline (TC) for wound dressing **(**Fig. 6c**)** [90]. Due to the using of antibiotic TC and the sustainable delivery ability of MCM-41, the created hydrogel relieved wound pain and promoted the wound healing.

#### Composite hydrogels

Besides the above-mentioned biomolecules that used for the fabrication of bioactive hydrogels, composite hydrogels are also widely used in the field of biomedicine [91]. In recent years, researchers have conducted in-depth studies on the preparation and functionality of composite hydrogels, which has continuously promoted the development of their applications [92].

**Fig. 7:**
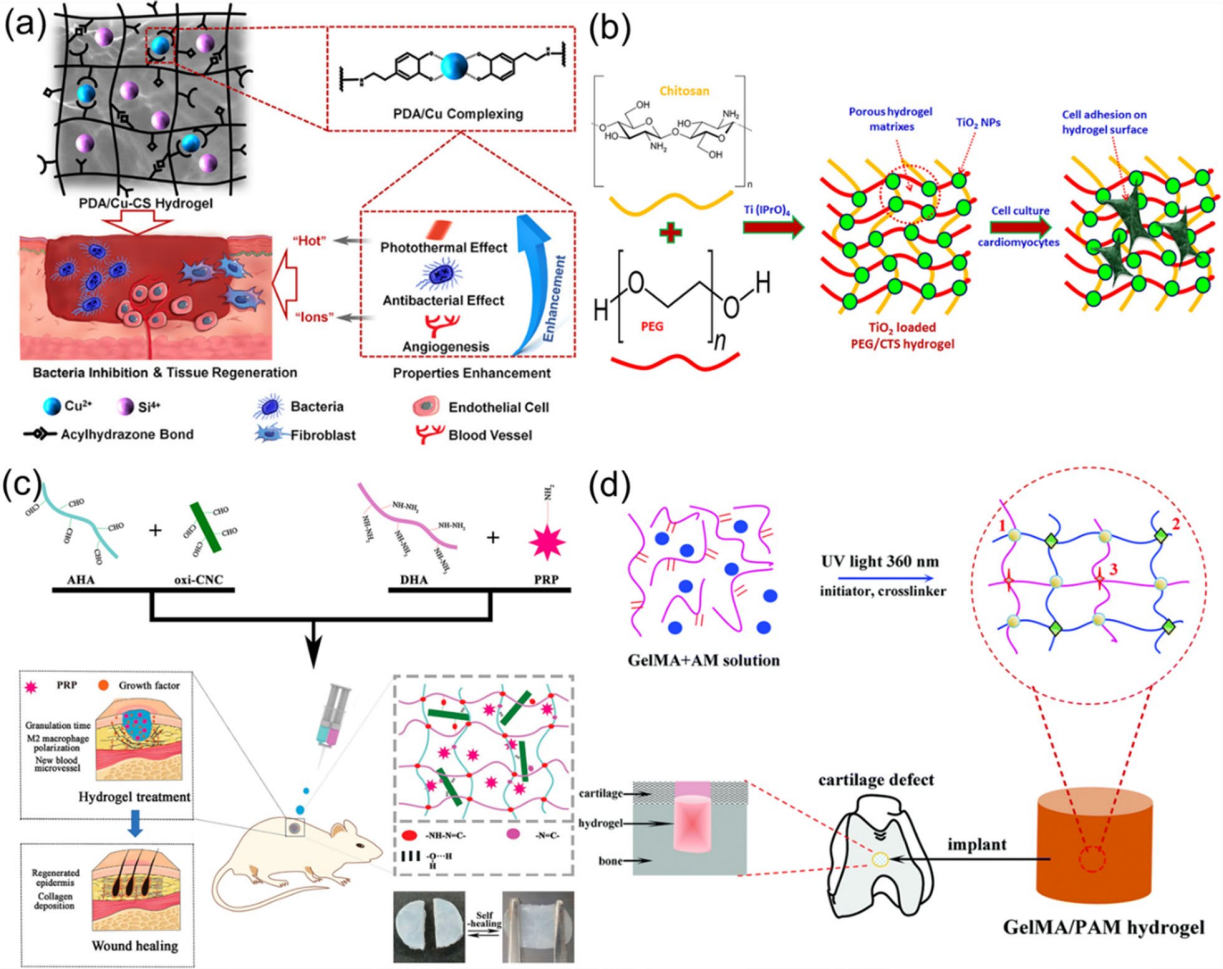
Synthesis and structure of other biopolymer hydrogels: **a** PDA/Cu-CS composite hydrogel. Reprinted from Ref. [93], Copyright 2020, American Chemical Society. **b** PEG/CTS hydrogels loaded with TiO_2_ NPs. Reprinted from Ref. [94], Copyright·2018, Elsevier. **c** Self-healing HA nanocomposite hydrogel. Reprinted from Ref. [95], Copyright·2022, American Chemical Society. **d** GelMA-PAM hybrid hydrogel. Reprinted from Ref. [96], Copyright 2017, Royal Society of Chemistry

Xu and co-workers reported the design and synthesis of functional hybrid polydopamine (PDA) hydrogel by conjugating PDA and copper-doped calcium silicate (Cu-CS), forming the PDA/Cu-CS composite hydrogel [93]. As shown in Fig. 7a, Cu-CS was synthesized using a sol–gel method, which further oxidized DA to PDA, while PDA complexed with Cu^2+^ that released from Cu-CS. The created hydrogel exhibited multiple functions, including the abilities of photothermal reaction, antibacterial ability, angiogenesis-mediation, cell proliferation, bio-adhesion, and self-healing. In another study, Liu et al. developed an injectable PEGylated-chitosan (PEG/CTS) hydrogel that loading with TiO_2_ NPs (Fig. 7b) [94]. The addition of TiO_2_ NPs into the PEG/CTS hydrogel improved its physicochemical and biological properties of the PEG/CTS hydrogel. The synthesized composite hydrogel exhibited improved compression modulus and better swelling performance, enhanced adhesion to cardiomyocytes, and tissue repair function. Therefore, their study provides a promising approach for the development of highly efficient patch repair materials for cardiac tissue with superior bioactivity and mechanical properties.

Composite hydrogels based on natural polymers have been widely used in the repair and regeneration of biological tissues due to their high similarity to the structures of biological tissue. Li et al. developed HA-based hybrid hydrogels using sodium hyaluronate and CNCs as the linking substrates, which showed sufficient strength and self-healing ability to accelerate skin wound healing [95]. As shown in Fig. 7c, aldehyde-modified sodium hyaluronate (AHA), hydrazide-modified sodium hyaluronate (ADA), and aldehyde-modified cellulose nanocrystals (oxi-CNC) were dynamically operated via a double-barreled syringe. The hydrazide bonds promoted the *in-situ* formation of hydrogels. Their study provides a good example for the development of drug-loaded self-healing hydrogels.

In another study that using hydrogels to repair biological tissues, Han et al. used methacrylic anhydride (MA) to chemically modify the Gel to obtain photo-cross-linkable GelMA, which was then further mixed with polyacrylamide (PAM) to form the GelMA-PAM composite hydrogel under the irradiation of UV light of 360 nm (Fig. 7d) [96]. The synthesized compisite hydrogel showed good mechanical properties and thermal stability, and could be applied for the cartilage repair in organisms. In addition, the in vitro cell culture tests have proved that the hydrogel had good biological activity and could promote the proliferation and growth of chondrocytes.

To make it more clear, the fabrication of bioactive hydrogels that used for SCI is described in detail, and the contents are summarized in Table 1.

**Table 1:** Bioactive hydrogels that prepared from different materials

Material	Name	Crosslinking methods	Application	Merit	Refs.
DNA	DNA–nSi Nanocomposite Hydrogel	Electrostatic Interactions	Drug delivery system for bone regeneration	Injectable, sustained-release therapeutic properties	[63]
	DNA-OA Hydrogel	Electrostatic Interactions,reversible imine linkages	Drug delivery system for bone regeneration	Sustained-release properties of the drug. Injectability	[64]
	AuNP-DNA hydrogel	Base complementation	Photothermal immunotherapy for tumors	Safety, injectability, biodegradability, ability to stimulate innate immunity	[65]
	CPT–DNA- hydrogel	Chemical crosslinking	Local Chemotherapy to Prevent Tumor Recurrence	Injectable, thermosensitive, and nuclease- and GSH-responsive properties	[66]
Protein	RSF-SNF hydrogel	Enzyme crosslinking	Tissue regeneration	Tunable mechanical properties	[72]
	Silk fibroin/collagen hydrogel	physically crosslinking	Tissue regeneration	Adjustable mechanical strength, good cell compatibility	[73]
	Silk-HA hydrogel	Enzyme crosslinking	Tissue engineering	Controllable gelation and degradation rates	[74]
	Bi_2_S_3_-BSA nanocomposite hydrogel	Chemical crosslinking	Photothermal therapy for tumors	Injectable and self-healing properties、 synthesis method is simple	[75]
	TA-PVA/BSA Hydrogel	Physically crosslinking	–	High mechanical strength and good water retention capacity, adjustable mechanical properties	[76]
	Mfp Hydrogel	Photochemical crosslinking	Cell adhesion and neurite growth	Supports adhesion and proliferation of multiple cell lines	[78]
Peptide	KK-BSA hydrogel	Chemical crosslinking	Promoting Infected Wound Healing	Rapidly degradable, injectable, self-healing, antibacterial and angiogenic ability	[81]
	photoactivated Fmoc-KDNBK hydrogel	Photochemical crosslinking	Cell culture	Controlled and fast sol–gel conversion	[82]
	Dentinogenic Peptide Hydrogel	Non-covalent interactions	Pulp tissue regeneration	Injectable properties, cytocompatibility, support DPSC proliferation and increase the potential for calcium phosphate deposition	[83]
	FK Hydrogel	Electrostatic Interactions	Drug delivery system	Controllable rate of drug release	[84]
Biomass polysaccharides	Alg/Gel/CNCs hydrogel	Intermolecular hydrogen bonding	Regeneration of bone tissue	Bio-ink in 3D-printing for tissue engineering	[87]
	TOCNFs CaPGO hydrogel	physically crosslinking	Regeneration of bone tissue	Injectable, inducing mineralization	[88]
	Chitosan-CMC-g-PF127 hydrogel	Chemical crosslinking	Diabetic wound healing	Mechanical properties, rheological properties, microporous structure, continuous drug releasee	[89]
	CMC / ZnO-MCM-41 hydrogel	hydrogen bonding	Wound healing and dressing systems	High mechanical properties, breathability, antibacterial	[90]
Composites	PDA/Cu-CS hydrogel	Electrostatic Interactions	Treatment of infectious wounds	Photothermal effect, antibacterial	[93]
	PEG/CTS hydrogel	Chemical crosslinking	Cardiac repair	More healthy and synchronous activity	[94]
	AHA/DHA/oxi-CNC hydrogel	Chemical crosslinking	Skin regeneration	High mechanical strength and self-healing ability, injectability	[95]
	GelMA/PAM biohybrid hydrogel	Chemical crosslinking	Chemical crosslinking	Cell adhesion, biocompatibility, degradable	[96]

### Functional regulation of bioactive hydrogels

In this section, the regulation of biological functions of hydrogels, including the cell differentiation, self-healing, anti-bacterial, injection, bio-adhesion, biodegradation, and other multi-functions, via various strategies are introduced and discussed.

#### Cell tissue behaviors

The speed of tissue repair is determined by the differentiation and regeneration of cells in the process of SCI. The differentiation and regeneration of spinal cord cells can be induced by adding GFs or bioactive drug molecules into the hydrogels. Especially in the process of vascular and nerve cell regeneration in the spinal cord, the good coating of hydrogels can guide the differentiation and regeneration of nerve cells in all directions. Because of its good infiltration, permeability, and biocompatibility, hydrogel plays an important role in the vascular regeneration, guiding the nerve differentiation, and promoting the cartilage formation [97, 98].

**Fig. 8:**
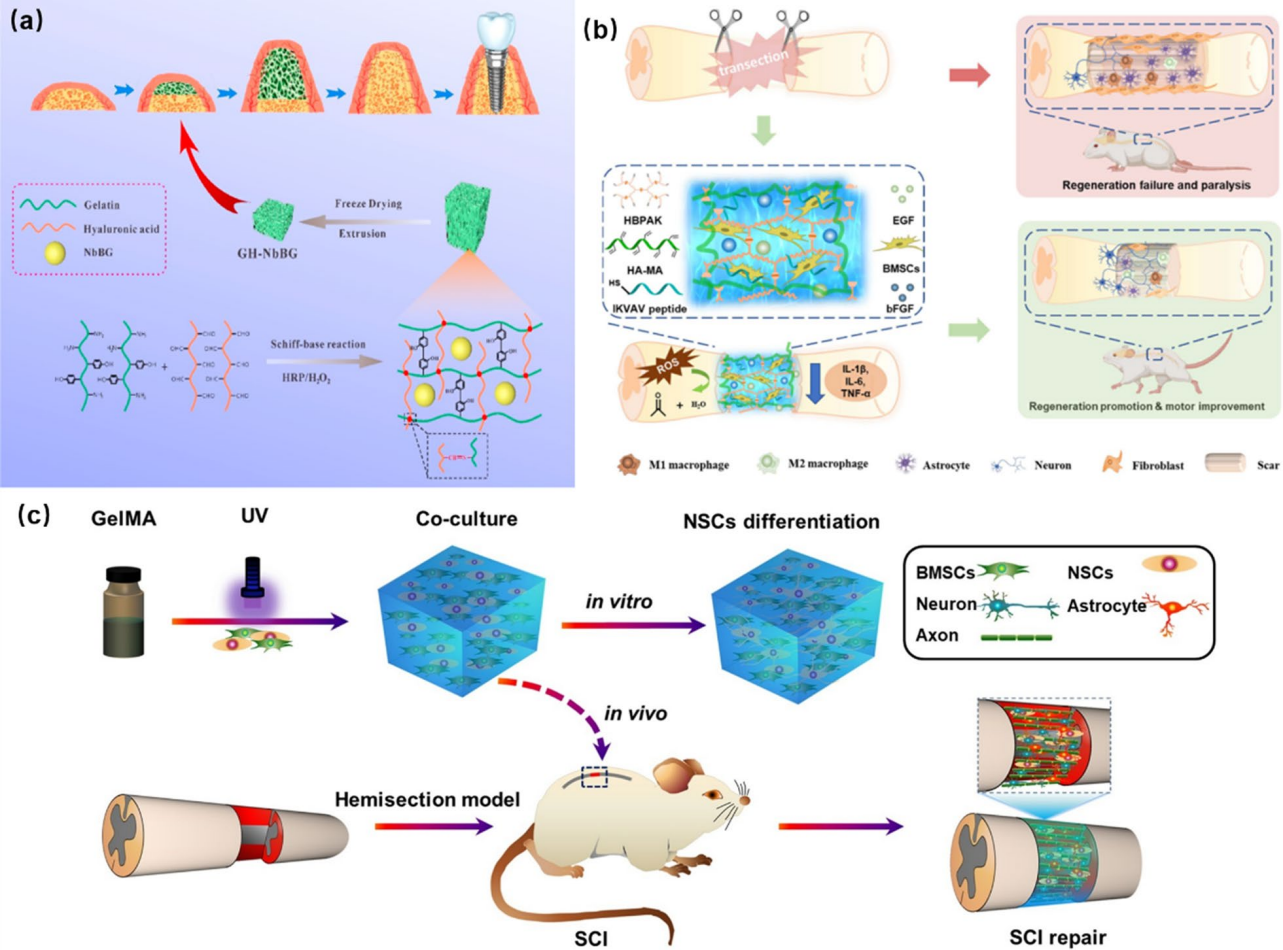
Cell biocompatibility of bioactive hydrogels: **a** GHNbBG hydrogel for osteogenesis. Reprinted from Ref. [99], Copyright 2023, Elsevier. **b** EGF and BFGF-loaded peptide hydrogels for SCI. Reprinted from Ref. [26], Copyright 2023, Elsevier. **c** GelMA hydrogel loaded BMSCs and NSCs for SCI repair. Reprinted from Ref. [103], Copyright 2020, Elsevier

The hydrogels with high mechanical strength have strong pressure-bearing capacity and swelling ability, which play a supporting role. Using this property of hydrogels, Zhao et al*.* developed a hydrogel with the ability of increasing bone mass through the self-expansion. In their study, gelatin-hyaluronic acid hydrogel (GH) was prepared by double cross-linking of oxidized hyaluronic acid (HA-CHO) and tyramine modified gelatin (GA-tyramine). A kind of swelling-enhanced GHNbBG hydrogel was prepared by adding niobium-doped bioactive glasses (NbBG) into the as-prepared hydrogel. The expansion of GHNbBG hydrogel was beneficial to the bone elevation and new bone was formed after the degradation of the hydrogel. Meanwhile, NbBG promoted the angiogenesis effectively in the process of hydrogel expansion (Fig. 8a) [99].

The cells in the sites of SCI are often accompanied by the inflammation. The reactive oxygen species (ROS) released by inflammatory immune cells will not only cause the apoptosis of normal cells around the spinal cord, but also inhibit the regeneration of neuro cells. Therefore, the removal of ROS that produced by inflammatory cells is also a very important strategy for the repair of SCI. For example, Li and co-workers proposed the synthesis of a hydrogel that can encapsulate the BMSCs and scavenge ROS [26]. As show in Fig. 8b, the neuro-specific peptide (IKVAV) is covalently linked to the hydrogel that formed by the cross-linking of hyperbranched polymer (HBPAK) containing thioacetal and methacrylate hyaluronic acid (HA-MA). Based on the good coverage and the flexibility of the formed hydrogel, the rat epidermal growth factor (EGF) and basic fibroblast growth factor (BFGF) were encapsulated only by physical methods. This kind of hydrogel could promote the polarization of M2 macrophages, protect BMSCs from the oxidation of ROS during the bone marrow interstitial transfer, and accelerate axonal regeneration.

In the preparation process of hydrogels that can be used for physiological tissue repair, the addition of therapeutic metal ions can accelerate the process of tissue repair and treatment [100, 101]. For example, in the work of Zhang et al*.*, the introduction of Mg^2+^ into the formed hydrogels not only regulated the cell behavior, but also promoted local bone tissue regeneration and repair [102]. There was a complexation between Mg^2+^ and acrylated bis-phosphonate (Ac-BP), which driven the co-assembly of Mg^2+^ and Ac-BP to form Ac-BP-Mg^2+^ NPs. The photo-initiator was added to the mixed solution of methacrylated HA (MeHA) and Ac-BP-Mg^2+^ NPs, to form hybrid hydrogels by the photo-induced stimulation. In physiological tissue, the hydrogels exhibited the ability to release Mg^2+^ continuously, resulting in enhanced performance for the bone regeneration and osteogenesis at the expected sites.

In the repair of SCI, the nerve repair is one of the important steps in the whole repair process. In the work of Zhou et al*.*, a hydrogel for spinal cord repair has been developed to reverse the differentiation of NSCs into astrocytes and to differentiate as many neurons as possible. As shown in Fig. 8c, gelatin methacrylamide (GelMA) hydrogels containing BMSCs (1 × 10^7^ mL^−1^) and NSCs (1 × 10^7^ mL^−1^) were synthesized through the photo-encapsulation. The formed GelMA hydrogels showed enhanced ability in vitro, and promoted the differentiation of NSCs into neurons in the in vivo SCI repair. Their results proved that the designed GelMA hydrogels loading with BMSCs and NSCs promoted neuronal differentiation and recovery of motor function significantly, which exhibited high application potential in the SCI repair to promote neuronal differentiation [103].

#### Self-healing property

Filling the SCI cavity with self-healing materials can provide bridges and carriers for the regeneration of NSCs, axons, and myelin sheath, and create channels for the transmission of electrical signals in the spinal cord. Therefore, the regenerative microenvironment created by the self-healing materials is beneficial to the repair of SCI [104, 105]. In the process of SCI repair, self-healing hydrogels can effectively avoid the damage and wear that caused by hydrogels in the transportation and harsh environment, and ensure the maximum value of hydrogels in the process of treatment through the ability of self-repair. Meanwhile, the hydrogels can better promote the repair of SCI [106, 107].

**Fig. 9:**
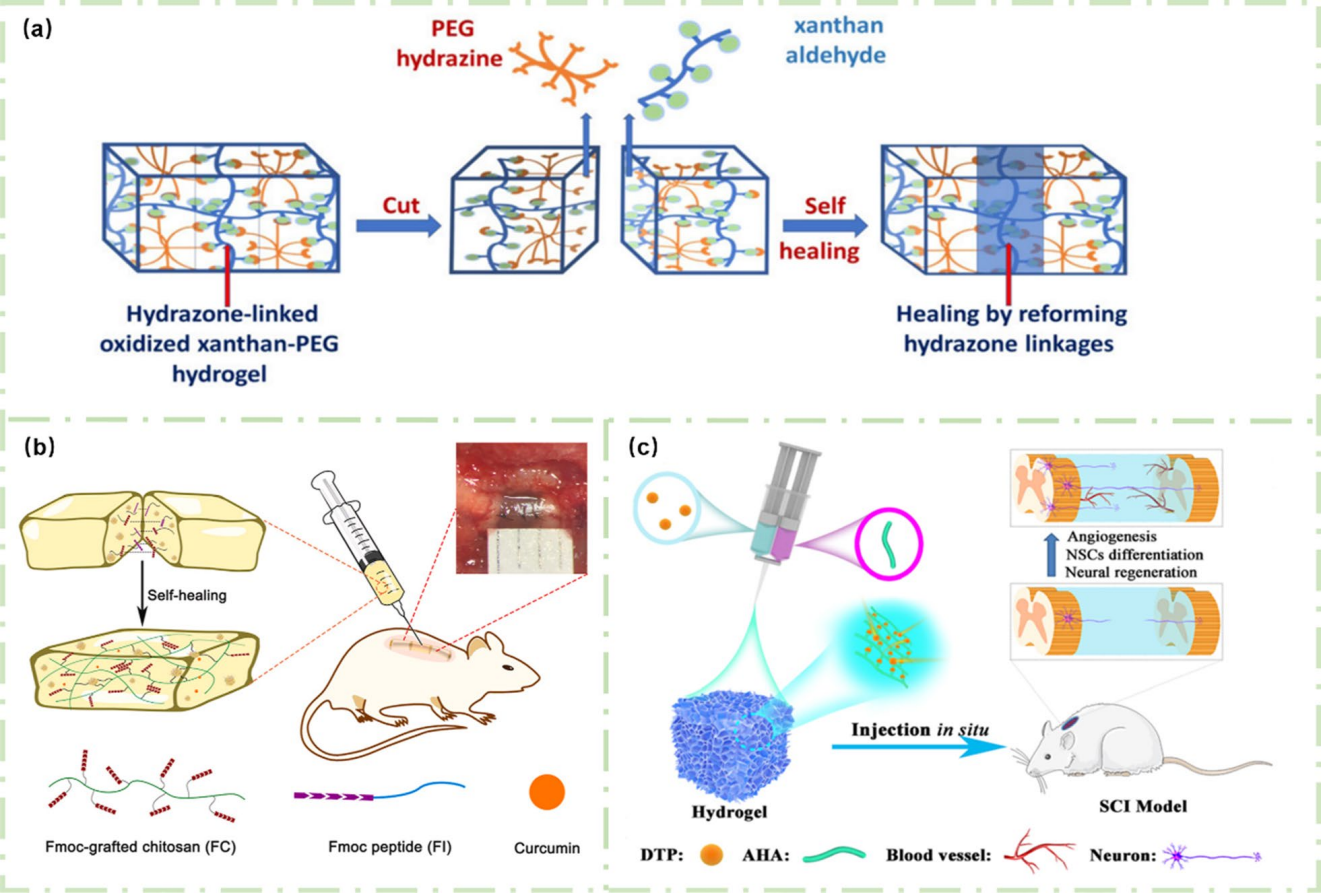
Self-healing hydrogels for SCI repair: **a** XG-PEG self-healing hydrogel. Reprinted from Ref. [108], Copyright 2018, American Chemical Society. **b** Self-healing FC/FI-Cur hydrogel for treating SCI. Reprinted from Ref. [109], Copyright 2021, Elsevier. **c** Self-healing AHA/DTP hydrogel for repairing SCI. Reprinted from Ref. [110], Copyright 2022, Elsevier

The self-healing process of hydrogels is often realized by dynamic chemical bonds. For example, a new type of xanthan gum-polyethylene glycol (XG-PEG) hydrogel was prepared by dynamic, pH-responsive, and biodegradable binding reactions in the work of Singh and co-workers [108]. As shown in Fig. 9a, under the action of dynamic covalent binding between PEG and XG, the created hydrogel exhibited excellent self-healing ability.

In the work of Luo et al*.*, the dynamic π-π interaction between benzene groups was used to obtain the self-healing ability of hydrogels [109]. Peptide IKVAV is a laminin-derived peptide that can promote the growth of axons in the spinal cord, and fluorenylmethoxycarbonyl (Fmoc) group contains three circular rings with a strong π-π interaction. The π-π interaction of the peptide chain is enhanced by modifying the Fmoc group at the end of the peptide molecule. As shown in Fig. 9b, the FC/FI-Cur hydrogel was synthesized by adding curcumin (Cur) into the Fmoc peptide (FI) and Fmoc-grafted chitosan (FC) during the co-assembly process [109]. The dynamic and reversible π-π interaction of the FC/FI-Cur hydrogel made the created hydrogel had good self-healing ability. More importantly, Cur coated with hydrogel could be released slowly and continuously, which helped to resist the inflammation at the sites of SCI and promoted the SCI repair.

In another study, Li and co-workers demonstrated the fabrication of self-healing AHA/DTP hydrogels by *in-situ* cross-linking of aldehyde-modified HA (AHA) and 3-methylithiobis (propionylhydrazide) (DTP) through double syringes (Fig. 9c). There are several dynamic covalent bonds in DTP, which can realize the self-healing of the synthesized AHA/DTP hydrogels. Meanwhile, the AHA/DTP hydrogels could bridge the injured sites of spinal cord and promote the healing and repair of spinal cord through their self-healing ability, creating a favorable microenvironment for the growth of nerves and axons to promote the functional repair of SCI [110].

#### Anti-bacterial and anti-inflammatory properties

Injured spinal cord is more prone to the infection due to the destruction of microenvironment and tissue exposure, which leads to other complications or slows down the repair and regeneration of SCI [111]. Therefore, the development of anti-inflammatory and anti-bacterial hydrogels for the repair of SCI is helpful to reduce the occurrence of various complications in the repair process [7]. In the preparation process of anti-bacterial SCI repair hydrogels, the addition of anti-bacterial factors can greatly improve the antibacterial activity of hydrogels. Chitosan (CTS), polydopamine (PDA), metal nanoparticles, as well as graphene and its derivatives all have good anti-bacterial properties, revealing potential importance for preparing functional hydrogels [112, 113]. For instance, Gallardo et al*.* successfully introduced PDA into guanosine-boric acid (GB) to form PGB hydrogel using 3D printing technology, which greatly increased the content of PDA in the hydrogel [114]. The fabricated PGB hydrogel exhibited obvious fiber network structure, and the incorporation of PDA greatly improved the osteogenic activity and biocompatibility of PGB. In addition, PGB hydrogel revealed good anti-bacterial activity. Compared with GB hydrogel alone, PGB reduced the bacterial adhesion and biofilm formation, and fundamentally inhibited the bacterial growth.

**Fig. 10:**
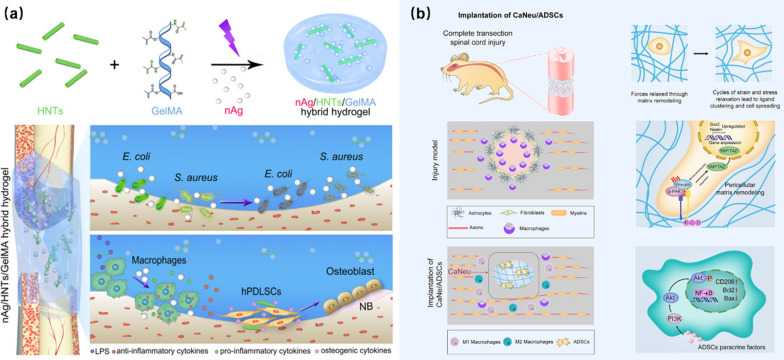
Anti-bacterial and anti-inflammatory properties of hydrogels: **a** nAg/HNTs/GelMA for preventing bacterial infection and promoting bone tissue regeneration. Reprinted from Ref. [115], Copyright 2020, Elsevier. **b** ADSCs-loaded CaNeu hydrogel for the formation of anti-inflammatory microenvironment. Reprinted from Ref. [116], Copyright 2021, Elsevier

In another case, Ou et al*.* reported the combination of the bone immunomodulatory and anti-bacterial ability of hydrogels for accelerated bone tissue regeneration. In their study, the silver nanoparticles/halloysite nanotubes/gelatin-methacrylic acid (nAg/HNTs/GelMA) hybrid hydrogel was prepared by the photopolymerization, as shown in Fig. 10a [115]. GelMA has a similar environment to natural extracellular matrix with good biocompatibility. nAg reveals excellent spectral anti-bacterial activity and low toxicity, and can show strong anti-bacterial and anti-inflammatory effects in the process of wound healing. Halloysite nanotubes (HNTs) is a kind of naturally occurring silicate nanotubes, which has great potential in drug transport and bone tissue regeneration. Due to the synergistic effects of all components, the injured spinal cord was tightly wrapped after the introduction of the nAg/HNTs/GelMA hydrogel into the injured sites. The existence of HNTs strengthened the electrostatic interactions between the hydrogel and nAg, which maintained long-term and comprehensive antibacterial activity. Meanwhile, HNTs regulated the bone immune system and promoted bone tissue regeneration. Therefore, the designed nAg/HNTs/GelMA hydrogel relieved the inflammation of the SCI sites greatly, prevented the bacterial infection effectively, and accelerated the repair of SCI.

SCI can produce a very serious inflammatory microenvironment, which will affect the cell survival and proliferation to reduce the efficiency of the repair of SCI. In the work of Yuan et al*.*, stem cells were used to enhance the adaptability and dynamics of the hydrogel and to repair SCI by reducing the microenvironment of injured sites [116]. As shown in Fig. 10b, cell-adaptable neurogenic (CaNeu) is developed as the carrier of adipose-derived stem cells (ADSCs) (1 × 10^7^ cells mL^−1^), and the CaNeu hydrogel loaded with ADSCs formed a dynamic permeable network and solved the problem of the apoptosis of ADSCs in inflammatory environment by inducing the polarization of macrophages to form an anti-inflammatory microenvironment. In a recent work [117], Li and co-workers developed a spinal cord hydrogel patch, which revealed good anti-bacterial, anti-inflammatory, and analgesic effects. The fabricated hydrogel patch inhibited the expression of tumor necrosis factor effectively, and the good biocompatibility expanded its broad applications in the SCI repair and the inhibition of postoperative infection.

#### Injectable ability

In the injured spinal cord, the cavity shape of the wound is usually irregular. The shape and strength of the hydrogels can satisfy the SCI of different traumatic depths, especially the injectability of the hydrogels can tightly fill the SCI cavity. Whether in the drug release, adhesion or promoting cell regeneration and other aspects can play a personalized treatment [118–120]. In addition, the injectable hydrogels are also more suitable for minimally invasive surgery, and the bioactive hydrogels with good fluidity and injectability can enter and infiltrate the injured sites through the syringe, which is beneficial to the repair of SCI in a easy way [121].

For instance, Zhou *and* co-workers synthesized a hydrogel using peptide and poly (ethylene oxide) diacrylate (PEGDA) by the *in-situ* Michael addition reaction [122]. First, the amino terminal of the peptide KYIGSRK was coupled with Ibuprofen to form the Ibuprofen-KYIGSRK, in which the lysine at both ends of the peptide connected two PEGDA polymer chains through the Michael addition reaction to form a PEGDA hydrogel network with injectable property. In the sequence of KYIGSRK, YIGSR promoted the cell adhesion and nerve terminal growth. The presence of Ibuprofen at peptide played an anti-inflammatory effect and promoted the regeneration of neurons. In addition, Ibuprofen combined with peptide reduced the random diffusion in the SCI sites due to their synergistic effects. This injectable hydrogel was synthesized by the *in-situ* reduction without adding any catalyst, showing the advantages of good biocompatibility, anti-inflammation, and controllable drug release, which provides a facile strategy and new idea for treating irregular and minimally invasive SCI.

**Fig. 11:**
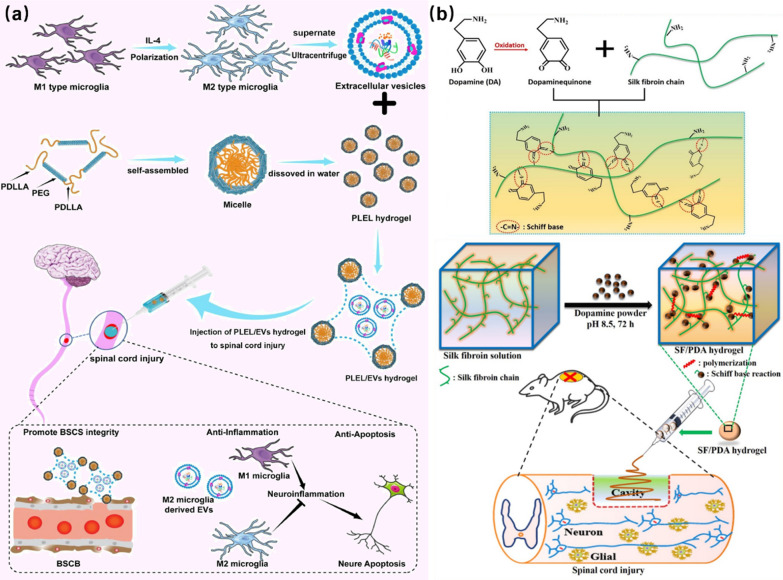
Injectable ability of hydrogels: **a** Injectable thermosensitive PLEL/EVs hydrogel for SCI repair. Reprinted from Ref. [8], Copyright 2022, Elsevier. **b** Injectable SF/DA hydrogel for SCI repair. Reprinted from Ref. [123], Copyright 2020, Elsevier

In the process of repairing SCI, the persistent inflammation is the root cause that hinders the cell regeneration, so solving the problem of the inflammation in the sites of SCI is helpful for rapid SCI repair. In the study of Wang et al., extracellular vesicles (EVs) were compounded into poly (d, l-liactide)-poly (ethyleneglycol)-poly(d, l-rellism) (PLEL) for the form of PLEL/EVs hybrid hydrogel. EVs microglia M2 can reduce the inflammation and promote the nerve regeneration, and the formed hydrogel was useful for solving the inflammation in the process of SCI repair. The synthesized PLEL/EVs hydrogel showed enough fluidity to enter the injured sites of the spinal cord through a syringe, as indicated in Fig. 11a. In addition, the PLEL/EVs bioactive hydrogel exhibited sensitive temperature response that can rapidly gelate and wrap the injured sites at body temperature, promoting the nerve regeneration and accelerating the SCI repair [8]. In another work, Chen et al*.* reported the design and synthesis of injectable SF/DA composite hydrogels by the auto-polymerization of silk fibroin (SF) and dopamine (DA). As shown in Fig. 11b, the fabricated SF/DA bioactive hydrogels had good injectability, which could be used as potential materials for the tissue adhesion, hemostasis, and other medical applications. Meanwhile, the addition of DA into hydrogels provided the possibility for the repair of SCI cells, and played a good role in promoting the axon growth and cell differentiation [123].

#### Biological adhesion

The complete covering of damaged tissues in the injured sites and the close contact with the broken end of nerves, blood vessels, and muscles are still big problems for repairing SCI [124]. Usually, the free repair material cannot attach and repair the injured sites well in the complex microenvironment of SCI. Therefore, the preparation of repair materials with certain adhesion ability to the SCI sites is a key step to promote the construction of repair and treatment of SCI [125, 126]. The good coating of bioactive hydrogels can achieve close contact with the SCI sites to accelerate the repair of the injured spinal cord. Through the modification of hydrogels to increase their biological adhesion ability, the injured spinal cord can be wrapped more closely, and can be repaired continuously and stably [127].

By enhancing the adhesion of hydrogels, it is possible to create a favorable environment for the proliferation, differentiation, and growth of cells in the injured spinal cord, which can effectively shorten the time of tissue repair and accelerate the speed of healing. For instance, Cai et al*.* successfully improved the adhesion and proliferation of NSCs in the spinal cord by the photo-fixation, which provided a good site for neuronal regeneration and produced neuronal tissue and speed up the repair of SCI [128]. The smooth surface of hydrogels is often difficult to provide the adhesion sites for cells or proteins. In the work of Staubitz et al*.* the problem of poor adhesion of hydrogels has been solved by adding the adhesion proteins into hydrogels [129]. The mercaptan of protein combined with the imide of poly (hydroxyethyl methacrylate) (pHEMA) through the Michael reaction, and the addition of protein into the hybrid hydrogels realized the biological functionalization of pHEMA and increased the biological adhesion ability of pHEMA hydrogels.

**Fig. 12:**
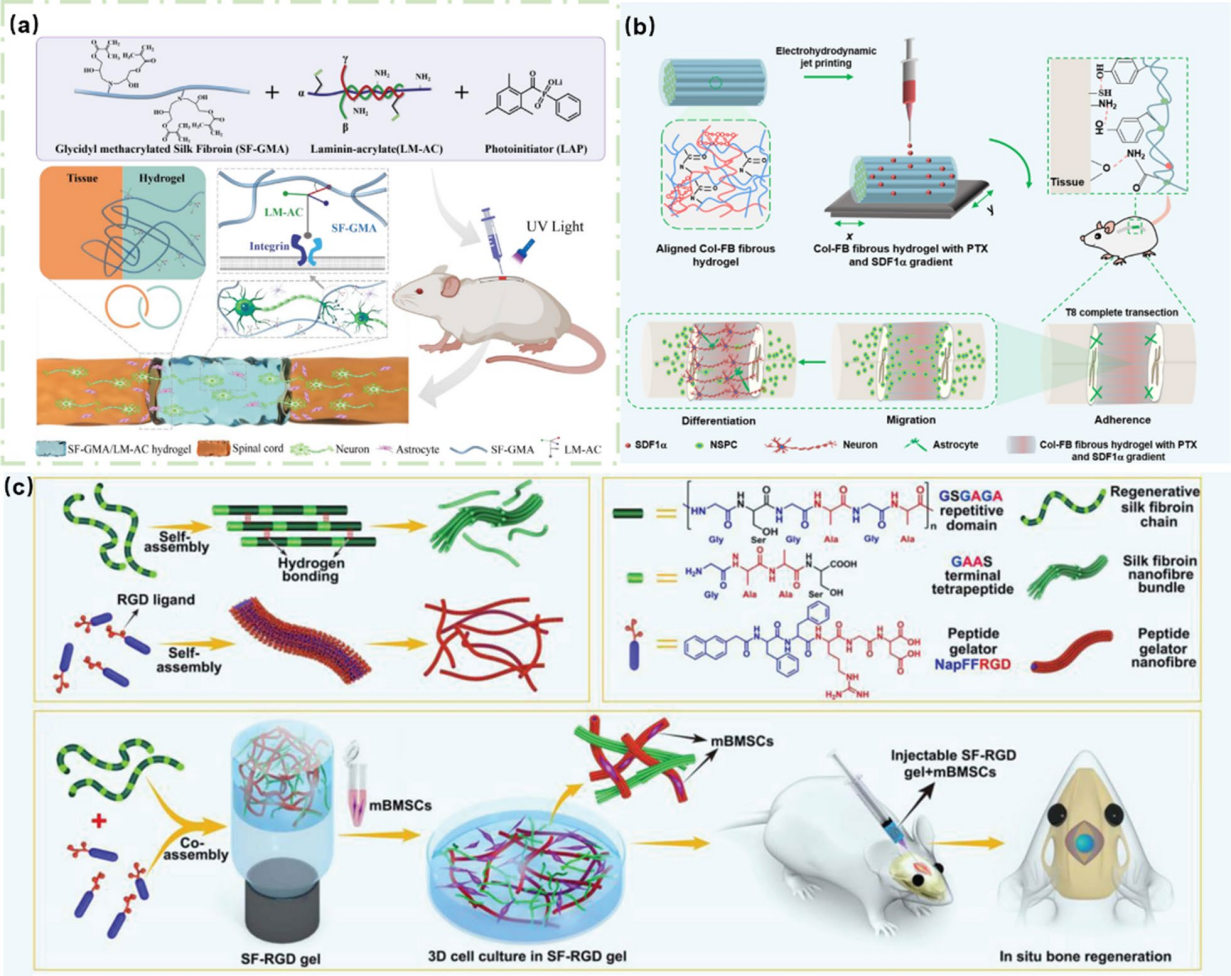
Biological adhesion of hydrogels: **a** SF-GMA/LM-AC hydrogel with high adhesion for SCI repair. Reprinted from Ref. [130], Copyright 2023, Elsevier. **b** Col-FB hydrogel for SCI repair. Reprinted from Ref. [131], Copyright 2022, American Chemistry Society. **c** SF-RGD hydrogel for the SCI adhesion and osteogenic differentiation. Reprinted from Ref. [132], Copyright 2019, Wiley–VCH

Liu and co-workers demonstrated a strategy to *in-situ* form bio-adhesive hydrogels at the SCI site. As shown in Fig. 12a, glycidyl methacrylated SF (SF-GMA), laminin-acrylate (LM-AC), and photoinitiaor (LAP) were injected into the SCI site, and the cross-linking ability of LAP was triggered by the UV light irradiation, forming a SF-GMA/LM-AC hydrogel network entangling with the spinal cord tissue and stably wrapping the SCI site. Different from other physical adhesions, the SF-GMA/LM-AC hydrogels revealed strong adhesion and infiltration to SCI sites, in which LM-AC promoted the differentiation and growth of spinal cord axons with enhanced biological activity of materials [130].

While ensuring the adhesion of the hydrogels, the elasticity and stretchability are also important for repairing SCI. In the work of Chen et al*.*, bioactive hydrogels with good stretchability and adhesion were prepared to deliver GFs and drugs for the SCI repair, which ensured close contact with the injured sites and promoted the differentiation of neurons and the repair of SCI [131]. As indicated in Fig. 12b, the repair process of the hydrogels was presented, in which the oriented collagen-fibrin (Col-FB) hydrogel with interacting network structure was prepared by electrospinning and *in-situ* sequential cross-linking method. The fibrin network had good elasticity, and the formed hydrogel exhibited enhanced mechanical properties after the conjugation with collagen. After that, stromal cell-derived factor-1α (SDF1α) and paclitaxel (PTX) were injected into the as-prepared Col-FB hydrogels by electrodynamic fluid jet printing technique to form a middle-to-both sides concentration gradient. It was found that the Col-FB hydrogels exhibited excellent adhesion and tightly connected the ends of SCI. The excellent tensile and mechanical properties of hydrogels ensured the lasting connection between Col-FB hydrogels and the injured sites. In addition, the concentration gradient of SDF1α and PTX in the Col-FB hydrogels showed continuous release in the injured spinal cord. Meanwhile, differentiated neurons migrated with the help of the Col-FB hydrogels and accelerated the repair of SCI nerve.

Previously, Yan and co-workers reported a new type of hydrogel as a biomimetic matrix to promote the cell proliferation and adhesion [132]. As shown in Fig. 12c, peptides containing RGD ligands were co-assembled with SF to form the SF-RGD hydrogels. The presence of RGD not only adhered the BMSCs to the hydrogels, but also realized the adhesion of hydrogels to the sites of SCI. Therefore, the designed bioactive hydrogels promoted the adhesion and proliferation of mBMSCs, and provided a biomimetic microenvironment for the osteogenic differentiation.

#### Biodegradation ability

Through the addition of biomolecules, such as dopamine, polyvinyl alcohol, hyaluronic acid (HA), and others into the preparation process of hydrogels, it is possible to synthesize hydrogels with good biocompatibility and biodegradation ability [133]. The biodegradable hydrogels can solve the problem of the direction of materials after the repair of physiological tissue. The degradable hydrogels can trigger their degradability through pH, heat, light, and other stimulations [134, 135]. In addition, the targeted release of drugs can be achieved through the degradation of hydrogels, and the accurate treatment of local damage can be achieved. Therefore, the development of biodegradable hydrogels is of great significance in the repair of SCI [136]. In the work of Shi et al*.*, a biodegradable PEG-based hydrogel was designed and synthesized. In vivo experiments in mice, the hydrogel could be degraded within 2–8 weeks and excreted through spleen and liver [137]. In another work, Xu et al*.* used the degradation of hydrogels to achieve drug release. The biodegradable hydrogel can be completely degraded in 7–8 weeks, and the drug can be released slowly in the process of degradation [138].

In the work of Xu and co-workers, PDA-modified germanium phosphide (GeP) nanoparticles (GeP@PDA) were incorporated into DA-grafted HA hydrogels (HA-DA) to prepare degradable hydrogels (HA-DA/GeP@PDA) with good electrical conductivity [139]. GeP@PDA formed a good electronic network path in the HA-DA/GeP@PDA system, which enhanced the electrical conductivity of the composite hydrogels. The synthesized hydrogels promoted the immune regulation, endogenous angiogenesis, and neurogenesis of neural stem cells.

**Fig. 13:**
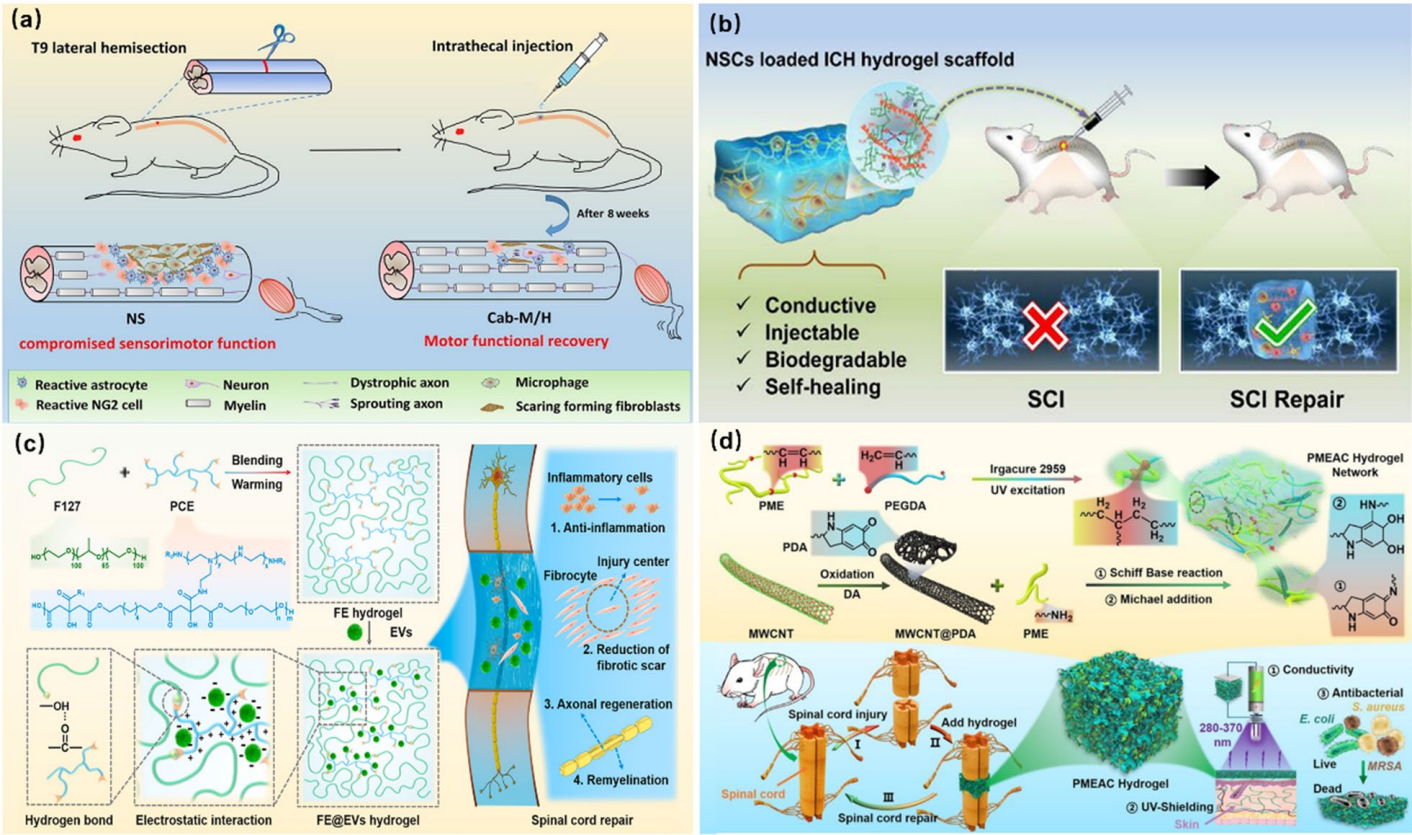
Multi-functions of bioactive hydrogels: **a** Biodegradable hydrogel Cab-M/H gels for healing injured site. Reprinted from Ref. [140], Copyright 2019, Elsevier. **b** Multifunctional conductive ICH/NSCs hydrogel for SCI repair. Reprinted from Ref. [142], Copyright 2023, American Chemistry Society. **c** Multifunctional FE/EVs hydrogel for promoting neuronal differentiation and axon formation. Reprinted from Ref. [25], Copyright 2021, Elsevier. **d** PMEAC hydrogel scaffold for regulating the microenvironment motor function recovery. Reprinted from Ref. [146], Copyright 2022, Elsevier

Li and co-workers also synthesized a biodegradable conductive hydrogel scaffold for the repair of SCI. The synthesized degradable hydrogel realized the sol–gel transformation under the control of temperature, which was beneficial to injection and *in-situ* gelation at the site of SCI. Based on this design, bioactive substances, cells, and drugs can be loaded into the hydrogels by simple injection. As shown in Fig. 13a, cabazitaxel (Cab)-loaded micelles (Cab-M) was mixed into thermosensitive hydrogels through *in-situ* synthesis. The Cab-M/H hydrogel was gelated *in-situ* at the site of SCI in mice. After 8 weeks of treatment, it was found that the injured site healed obviously and the Cab-M/H was degraded. The presence of Cab effectively promoted the growth of neurons. In addition, degradable Cab-M/H revealed less invasiveness and could continuously release Cab to achieve effective SCI repair [140].

#### Multi-functions and coordination

Usually, the microenvironment of SCI is very complex, so various factors should be considered in the process of the SCI repair, such as the inflammation, nerve repair, cell regeneration, antibacterial, tissue healing, and others [141]. The hydrogels with single treatment parameter are difficult to achieve satisfactory repair effect, and it is a very potential treatment method for the comprehensive regulation and treatment of the microenvironment in SCI [15]. For instance, in the work of Liu et al*.* conductive hydrogel scaffolds (ICH/NSCs) loaded with exogenous NSCs were assembled with amino gelatin (NH_2_-Gelatin) and aniline tetramer grafted with oxidized hyaluronic acid (AT-OHA). As shown in Fig. 13b, the ICH/NSCs showed good injectability and electrical signal conduction, which effectively induced the differentiation of NSCs and inhibited the formation of scar tissues. At the same time, the good degradability and self-repair ability also accelerated the efficiency of the SCI repair [142].

Mesenchymal stem cells (MSCs) can promote the repair of SCI by guiding neuronal differentiation, inhibiting the scar tissue formation and promoting the axon growth [143]. EVs derived from MSCs can improve the spinal cord microenvironment through mimicking cell paracrine secretions and have a better regulatory effect than MSCs [144, 145]. Therefore, Wang et al*.* used MSCs-derived EVs instead of MSCs transplantation to regulate the microenvironment of SCI to promote cell regeneration and differentiation. In order to achieve long-term preservation and controlled release of EVs in SCI tissue, an anti-inflammatory F127-polycitrate-polyethyleneimine (FE) hydrogel with cell adhesion and injectability was developed. FE hydrogel achieved long-term and sustainable release of EVs into the spinal cord [25]. As shown in Fig. 13c, F127 and polycitrate-polyethylene glycol-polyethyleneimine (PCE) were connected by hydrogen bonds between polymers to form FE hydrogels. Positively charged PCE was then combined with EVs to form the FE/EVs hydrogel network through electrostatic interactions. Due to the good adhesion, the FE/EVs hydrogel could be inject into the SCI sites to form a dense package. With its good biocompatibility, FE promoted skeletal muscle regeneration and inhibited the production of inflammation in the injured environment, but also provided a good carrier for EVs. Therefore, the designed FE/EVs hydrogel was useful for controlling continuous release of EVs, promoting neuronal differentiation and axon formation, and contributing to the recovery of motor function. In this case, the FE/EVs hydrogel exhibited the advantages of good injectability, anti-inflammatory activity, high adhesion, and regeneration ability, which promoted the repair of SCI effectively.

In the injured spinal cord, the loss of the electrical signal transmission is one of the important factors to inhibit the spinal cord regeneration. For this reason, Wang et al*.* developed a multi-functional polycitrate-based nanocomposite (PMEAC) hydrogel scaffold, which had biomimetic mechanical and electrical properties of the spinal cord and could enhance the transmission of electrical signals to the injured spinal cord to promote the repair and regeneration of the spinal cord. As shown in Fig. 13d, the PMEAC hydrogel was prepared by simple self-crosslinking method using poly (citric acid-maleic acid)-ε-polylysine (PME) and multi-walled carbon nanotubes (MWCNTs) as precursors. The created PMEAC hydrogel scaffolds revealed multi-functional properties, such as the injectability, self-healing, tissue adhesion, broad-spectrum antibacterial properties, and UV light shielding. The transmission channel of electrical signal was built for the SCI, which was beneficial to the repair of motor nerve and the recovery of motor function. In addition, the natural antibacterial activity of polylysine could effectively resist the invasion of bacteria and reduce the occurrence of inflammation in the injured spinal cord. Therefore, the PMEAC hydrogel scaffolds regulated the microenvironment of the nerve regeneration by inhibiting inflammatory response and anti-bacterial activity, which effectively promoted the motor function recovery and myelin/axon regeneration after SCI. It is a safe and effective biomimetic electrical signal recovery strategy to promote the SCI repair and regeneration [146].

To make it more clear, in this part, the applications of functional hydrogels in SCI repair are described in detail, and the contents are summarized in Table 2.

**Table 2:** Applications of functional hydrogel in several-year-old restoration

Functional	Matrix	Additive	Methods	Model	Application	Refs.
Cell biocompatibility	HA-CHO, GA-tyramine	NbBG	Double cross-linking	Human umbilical vein endothelial cells (HUVECs)	Bone elevation to promote regeneration	[99]
	IKVAV, HBPAK, and HA-MA	EGF, BFGF	Physical package	Schwann cells	Promote the polarization of M2 macrophages	[26]
	Ac-BP	Mg^2+^	Photo-crosslinking	Human MSCs (hMSCs)	Release of magnesium ions to regulate cell behavior	[102]
	GelMA	BMSCs, NSCs	photo-encapsulation	Sprague–Dawley (SD) rats (200 − 250 g)	Promote stem cell differentiation and motor nerve repair	[103]
Self-healing property	PEG	Xanthan aldehyde	Hydrazone-link	NIH-3T3 cells	Self-healing	[108]
	Fmoc peptide, Fmoc-grafted chitosan	Curcumin	Co-assembly	Sprague–Dawley rats (1–3 days, P1)	Slow release of curcumin	109]
	aldehyde-modified AHA,	3-methylithiobis (propionylhydrazide)	In-suit cross-linking	Spinal tissue removed rats	Create a microenvironment suitable for nerve and axon growth	[110]
Anti-bacterial and anti-inflammatory properties	Guanosine-boric acid	PDA	3D printing	rMSCs	Reduce bacterial adhesion and biofilm formation	[114]
	GelMA	nAg, and halloysite nanotubes	Photopolymerization	Sprague–Dawley rats (200–250 g)	Relieve inflammation and inhibit bacterial infection	[115]
	Cell-adaptable neurogenic	ADSCs	Host–guest crosslinking	Sprague–Dawley rats (200–220 g)	Promote the polarization of M2 macrophages	116
Injectable ability	poly (ethylene oxide) diacrylate	Ibuprofen-KYIGSRK	in-situ Michael addition reaction	Sprague–Dawley rats	Concentrated release of ibuprofen	[122]
	PLEL	EVs	High temperature gelation	Rat T9 spinal cord clip model	Promote nerve regeneration and accelerate SCI repair	[8]
	SF	DA	Co-assembly	Primary hippocampal neuron	Tissue adhesion and hemostasis	[123]
Biological adhesion	Poly (hydroxyethyl methacrylate)	Adhesion proteins	Michael reaction	10% foetal bovine serum	Adhesion enhancement	[129]
	Silk Fibroin	Laminin-acrylate, and photoinitiator	ultraviolet irradiated cross-link	NSC	Differentiation and growth of spinal cord axons	[130]
	Collagen-fibrin	STromal cell-derived factor-1α, PTX	electrospinning and in-situ sequential cross-linking method	Sprague–Dawley rats (200–220 g)	Lasting connection to the injured site and continuity of nerve terminals	[131]
	SF	RGD,	co-assembly	Mice Calvarial Defect Model	Promote the adhesion and proliferation of BMSCs	[132]
Biodegradation	HA-DA	PDA, GeP, and DA	HRP/H2O2 triggers gelation	Sprague–Dawley rats	Good electrical conductivity	[139]
	PEG	poly(lactic acid)	Covalent connections	–	dual-drug release	[138]
Multi-functions and coordination2	NH_2_-Gelatin	ICH/NSCs, AT-OHA		Sprague–Dawley rats (250–350 g)	Induction of differentiation of NSCs and inhibition of scar tissue	[142]
	F127-FE	EVs	Electrostatic interaction	female rats (~ 230 g)	Promote neuronal differentiation and axon formation	[25]
	MWCNTs	PME	Self-crosslinking	Sprague–Dawley rats	Promote motor function recovery and myelin/axon regeneration	[146]

### Material-based bioactive hydrogels for SCI repair applications

In this section, the regulation of biological functions of hydrogels, including the cell differentiation, self-healing, anti-bacterial, injection, bio-adhesion, biodegradation, and other multi-functions, via various strategies are introduced and discussed.

#### Growth factors/drugs-loaded hydrogels for SCI repair

Among the available biomaterials for the SCI repair, injectable hydrogels offer enormous advantages. It can act as an ECM at the damaged sites, provide a 3D scaffold for the cell proliferation and migration, and provide a suitable local microenvironment for the nerve tissue regeneration [147–149]. In recent years, the use of neurotrophic factors in the repair of SCI has received considerable attention, but the barriers in the delivery of such factors remain unresolved. Hassannejad et al*.* designed an amphiphilic peptide hydrogel for the delivery of brain-derived neurotrophic factor (BDNF) [150]. This hydrogel achieved a controllable and slow release of BDNF within 21 days while retaining the biological activity of BDNF, which solved the common problems in the deliveryof GFs. In addition, the hydrogel self-assembled by amphiphilic peptides containing the IKVAV sequences, which effectively promoted the neurite outgrowth and induce the cell function and neural tissue regeneration. The obtained results showed that 6 weeks after implantation of the functionalized hydrogel, the injury site not only did not produce an inflammatory response, but also attenuated astrogliosis after SCI, enhanced axonal preservation, and provided a permissive environment for the cell migration and growth.

**Fig. 14:**
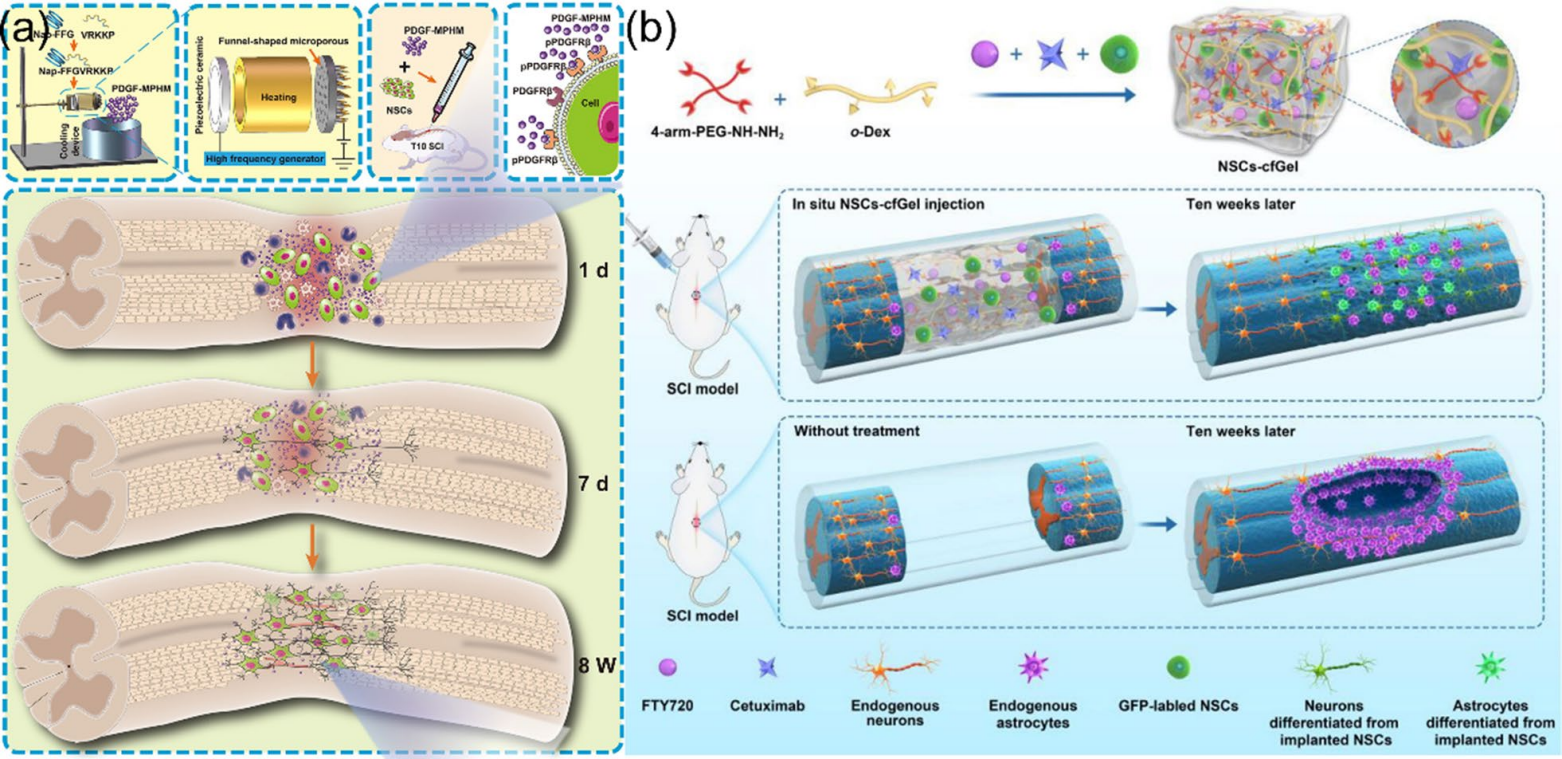
Growth factor/drug-hydrogel for SCI treatment: **a** PDGF-MPHM + NSCs hydrogel for SCI repair, Reprinted from Ref. [152], Copyright 2023, American Chemical Society. **b** Dual-drug NSCs-cfGel system for repairing SCI. Reprinted from Ref. [10], Copyright 2022, Elsevier

Alizadeh and co-workers designed a CTS-based injectable hydrogel. In their work, nerve growth factor (NGF)-overexpressing mesenchymal stem cells (hADSCs) (1 × 10^5^ cells mL^−1^) were encapsulated in chitosan/β-glycerophosphate/hydroxyethyl cellulose (CTS/β-GP/HEC) hydrogel [151]. Meanwhile, hADSCs and hADSC-encapsulated hydrogels were injected into the mice alone, and the recovery of the injured parts of the mice was observed. Studies have shown that hydrogels, as a 3D scaffold, effectively inhibited the migration of hADSCs, promoted the expression of NGF, and provided a suitable local microenvironment for the survival and proliferation of hADSCs, thus effectively improving the recovery of motor function in mice. In another similar case, Wu et al*.* developed injectable peptide-based hydrogel microspheres for loading and delivering platelet-derived growth factor BB (PDGF-BB) to injuried sites (Fig. 14a). PDGF-BB mimic peptide hydrogel not only had the advantages of good biocompatibility and high water-content, but also effectively activated PDGF receptor β and promote the axon regeneration [152]. In addition, the hydrogel maintained the proliferation of NSCs, protected neurons, and improved inflammatory response in the presence of myelin extract. Besides, Xu et al*.* utilized decellularized tissue matrix (DTM) as a natural biomaterial for spinal cord repair to prepare spinal cord-derived DTM hydrogel (DSCM) for spinal cord repair in piglets. Studies have found that DSCM retains many specific ECM components of the natural spinal cord, and its hydrogel has a nanofibrous structure that mimics ECM, providing a regeneration-promoting microenvironment for the treatment of spinal cord injuries. The results demonstrated slightly improved functional recovery at the site of spinal cord injury in just four weeks after DSCM hydrogel treatment. Therefore, DSCM hydrogel can be used as a microenvironment mimicking ECM to promote the enrichment, proliferation and differentiation of NSPCs [153]. Nonhuman primates and humans share many features in terms of neural architecture and organization of physiological processes, so nonprimate models of SCI can provide predictions of the safety and application potential of human SCI repair treatments. In the work of Rao et al*.* chitosan hydrogel was used as a bioactive material matrix to load neurotrophic factor 3 (NT3) to achieve slow and sustained release of neurotrophic factor into the environment after implantation. In a therapeutic experiment in a rhesus monkey spinal cord hemisection SCI model, it was found that the implantation of NT3-chitosan hydrogel was able to induce robust and robust axon regeneration, and robust long-distance axon regeneration was also observed. In addition, the enhanced neurogenesis and formation of nascent relay neural networks by endogenous stem cells were also involved in the recovery of sensory and motor functions, and the anti-inflammatory function of chitosan inhibited secondary lesions. The synergistic effect of NT3 and chitosan may be the key to promote the robust regeneration of axons [154].

Reducing the formation of glial scars and promoting neuron regeneration are effective ways to break through the treatment of SCI [155, 156]. In addition to GFs, nerve regeneration and protective drugs are also commonly used for the repair after SCI, such as Cur and minocycline hydrochloride (MH), chondroitinase ABC, and VX-210 (Cethrin). In the work of Nazemi et al*.*, they constructed a hydrogel for dual-drug delivery. Nerve regeneration drug PTX was encapsulated into polylactic acid-glycolic acid microspheres and embedded into hydrogel simultaneously with neuroprotective agent MH [157]. Their study showed that the slow sustained release of two drugs over eight weeks, the dual drug-loaded hydrogel treatment system effectively suppressed the inflammatory response of damaged tissue, increased the neuronal regeneration, and reduced the degree of fibrotic scarring. A reduction in scar tissue was observed after 28 days, suggesting potential performance for SCI treatment.

To address the tissue regeneration in fully cross-sectional SCI, Qi et al*.* developed another therapeutic system with dual drug delivery [10]. Two drugs, cetuximab and FTY720, were combined with NSCs (NSCs-cfGel) and delivered to the damaged area via an injectable hydrogel (Fig. 14b). The results showed that in this therapeutic system, two drugs synergistically promoted the proliferation and neuronal regeneration differentiation of NSCs, and reduced the cell differentiation around the damaged area. In turn, it reduced the formation of bruises, promoted the reconstruction of the nerve fiber network, and also played a positive role in improving the recovery of hindlimb movement. This combination of the therapy and injectable hydrogel delivery system provides a reliable idea for the repair of complete SCI *in-situ* [10]. In another case, Wang and co-workers reported a combined therapeutic system for multi-drug delivery to treat SCI [158]. Docetaxel (DTX) was combined with GFs to achieve the function of targeting damaged cells in the spinal cord. To improve the drug's ability to penetrate the blood-spinal cord barrier (BSCB), liposomes were further added to a solution of a novel cold heparin-modified poloxamer (HP) with highly specific binding to acidic fibroblast GF. The *in-situ* self-assembly induced the formation of hydrogels with 3D network structure in response to temperature, which not only had the function of targeted delivery of various drugs, but also realized the controllable release of drugs, improved the local microenvironment, and promoted the reconstruction of ECM. The hydrogel was beneficial for the axon regeneration, and conducive to the recovery of signal conduction, providing an effective way for the clinical treatment of SCI.

#### Polymer-modified hydrogels for SCI

In the SCI repair, a hydrogel system with excellent mechanical properties and good rheology fills the post-injury cyst cavity and supports the cell migration and axon outgrowth by replicating the complex structure of ECM [159]. Polymer systems with excellent rheological properties have received widespread attention, which can achieve the transition between sol and gel states in response to the stimuli or shear force, thereby being able to fill irregular and multi-shaped cavities. This method is more direct and effective than direct implantation of hydrogel [160]. In addition, this responsive hydrogel can also be used as a carrier to deliver therapeutic agents to promote rapid repair of SCI.

Thermosensitive hydrogels of poly(N-isopropylacrylamide) (PNIPAAm) exhibited good potential for the SCI repair due to their ability to undergo phase transition at a lower critical solution temperature [161, 162]. Based on the temperature phase transition ability of PNIPAAm, Bonnet et al*.* added PEG to increase the hydrophilicity of the hydrogel. The physically cross-linked copolymer was injected into the area of the SCI, and within minutes the copolymer gels formed a hydrogel. The PNIPAAm-g-PEG hydrogel did not induce significant inflammatory response in vivo and exhibited significant locomotor improvement [163]. In another case, Zhang et al*.* reported a thermosensitive poly(D,L-lactide)-poly(ethylene glycol)-poly(D,L-lactide) (PDLLA-PEG-PDLLA) hydrogel as a carrier of EV that derived from M2 microglia, which provided sufficient fluidity for hydrogel injection [8]. After entering the body, it quickly gelated with the increase of temperature. This thermosensitive hydrogel system slowly and continuously released loaded EVs at body temperature, thereby inducing the M2 polarization to reduce local inflammation and promote BCCB repair. In another case, An et al*.* successfully prepared hydrogels combined with natural polysaccharide agarose and PNIPAAm with thermosensitive function [164]. Au NPs and bone marrow stem cells (MSCs) (3 × 10^5^ cell mL^−1^) were embedded into the hydrogel matrix, and the incorporation of Au NPs and the porous morphology of the composite hydrogel significantly improved the cell proliferation and adhesion, contributed to autoneural regeneration and suppressed inflammatory responses, and played an active role in postoperative sports recovery.

Compared with chemically cross-linked hydrogels, polymer hydrogels cross-linked by physical action not only have excellent biocompatibility, but also do not produce the inflammation during SCI repair. It can also exhibit unique self-healing properties and shear-thinning behavior, which undoubtedly provides an attractive strategy for injectable SCI repair hydrogels [165–167]. Luo et al*.* reported an injectable, self-healing, conductive polymer hydrogel for enhanced tissue repair after SCI. Borax-functionalized oxidized chondroitin sulfate (BOC), BOC-doped polypyrrole (BOCP) and gelatin (Gel) were mixed under physiological conditions. Negatively charged BOC and positively charged BOCP were closely connected by electrostatic interactions, and the amino group in the Gel was conjugated with the aldehyde group in BOC to form a reversible Schiff base bond [168]. The presence of such dynamic covalent cross-links and non-dynamic covalent cross-links endowed the hydrogel with excellent self-healing and injectable properties. In addition, the in vivo experiments demonstrated that the presence of polypyrrole (Ppy)-enabled BOCPG hydrogel to act as a conductive bridge to promote endogenous neural stem cell migration and neuronal differentiation. In addition, it induced the regeneration of myelinated axons to the injury sites by activating the PI3K/AKT and MEK/ERK pathways, and promoted the repair of the SCI sites.

**Fig. 15:**
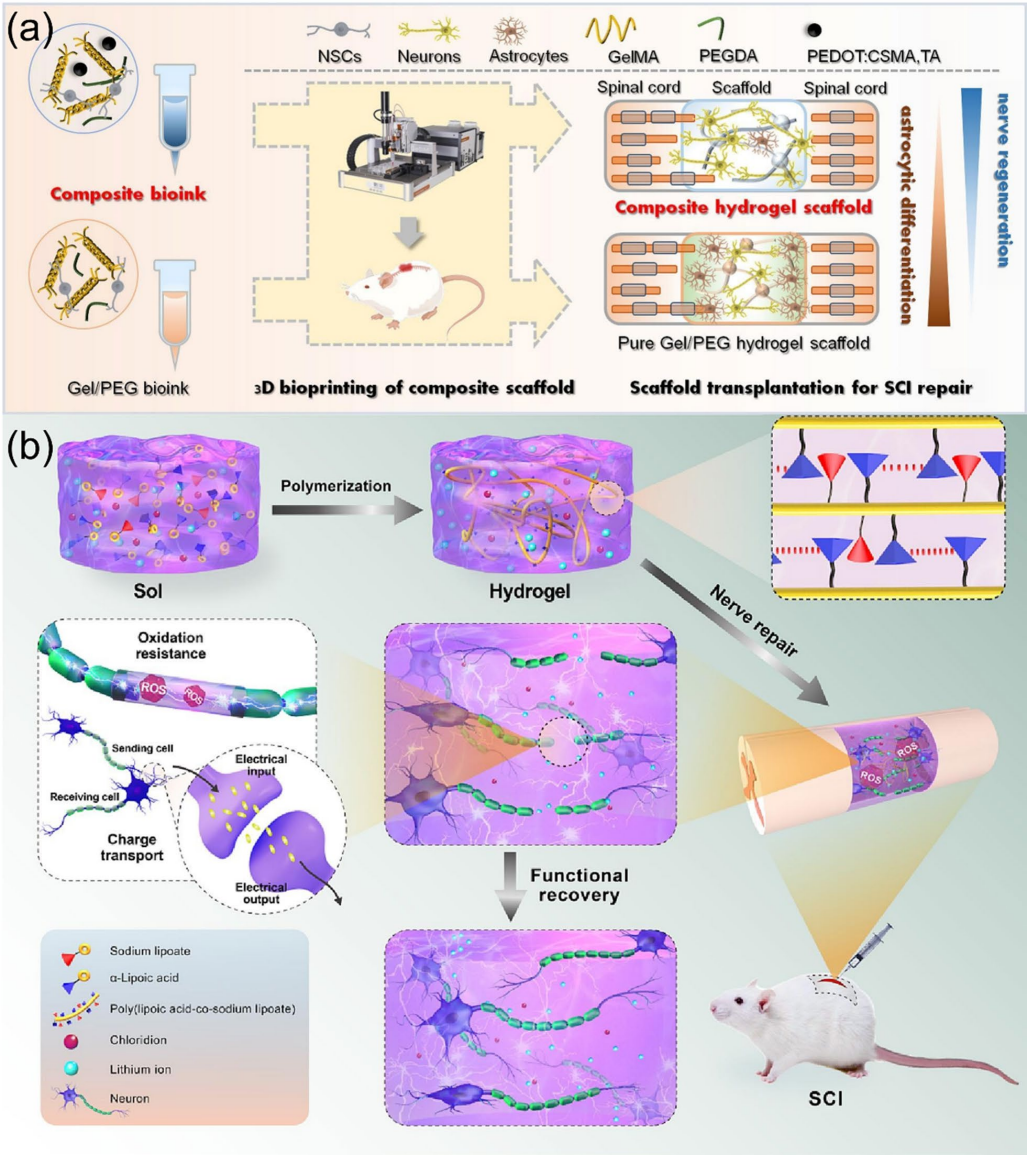
Polymer hydrogels for SCI repair: **a** 3D bioprinted conductive composite hydrogel for SCI repair. Reprinted from Ref. [169], Copyright 2023, Elsevier. **b** PLL hydrogel and its potential application in SCI repair. Reprinted from Ref. [174], Copyright 2023, Elsevier

To increase the rheological properties of 3D printing materials, Song et al. developed a composite bioink with good shear thinning and self-healing properties. In the synthesis process, the i*n-situ* redox polymerization of 3,4-ethylenedioxythiophene (EDOT) was carried out to form PEDOT in the presence of chondroitin sulfate methacrylate (CSMA) and tannic acid (TA)-doped with gelatin methacrylate (GelMA), which was then mixed with PEGDA to form a precursor solution for the synthesis of the Gel/PEG bioink (Fig. 15a). The 3D hydrogel scaffold printed with this bioink provided a good biological microenvironment for NSCs [169]. Its good electrical conductivity inhibited astrokeratinocyte generation in the scaffold effectively, providing a promising strategy for fabricating engineered neural tissue scaffolds.

In addition, some natural small molecules have excellent antioxidant capacity and can eliminate ROS in cells, and polymer hydrogels prepared with them as components also have excellent SCI repairing capacity [170, 171]. TA is a natural polyphenol present in many plants with excellent antioxidant and ROS scavenging properties [172]. In addition, TA can form a rich hydrogen bond network with various polymers to form elastic hydrogels through physical cross-linking. In the work of Wang et al*.*, the phenolic groups of TA were connected with the oxygen atoms in the polyethylene oxide (PEO) chains of Pluronic F-127 (PF127) through hydrogen bonding interactions. Therefore, the two components formed a viscous and elastic gel structure after mixing [173]. This hybrid hydrogel based on natural small molecules and polymers exhibited good sealing and anti-oxidation properties, which effectively reduced the generation of ROS, thereby inhibiting the inflammatory response.α-lipoic acid (LA) is a natural antioxidant capable of forming polymers through the ring-opening polymerization. Conductive poly (lipoic acid-co-lipoic acid sodium) (PLL) hydrogels were prepared by one-pot ring-opening polymerization using LiCl as a conductive filler (Fig. 15b) [174]. When the molar ratio of LA to sodium lipoate (SL) was 1:0.475, the formed hydrogel revealed a loose and porous 3D network structure, which could accelerate nutrient penetration and promote the cell growth. This PLL hydrogel with good adhesion and conductivity could effectively prolong the retention time of implanted materials, promoted electrical signal transfer, reduced oxidative stress, regulated inflammation, inhibited the glial scar formation, promoted the axon growth and rebuild synaptic structure, have shown great application potential for the repair of damaged tissues. In addition, Sofloud et al*.* exploited the Schiff-base bonds and particle interactions to develop an antioxidative and conductive hydrogel composed of polyaniline-grafted gelatin, oxidized alginate, and polyethyleneimine [175]. The composite hydrogel exhibited excellent electrical conductivity and antioxidant activity, could effectively induce the neural differentiation and promote the tissue repair. In addition, the physical properties of the hydrogel can be changed by adjusting the ratio of components, so as to achieve the purpose of injectability.

#### NP-functionalized hydrogels

Stem cell transplantation using biomaterial hydrogel scaffolds can serve as a very promising strategy for the SCI repair. Bioactive hydrogels have excellent biocompatibility, which serve as a bridge between injured tissue and normal tissue to provide a suitable microenvironment for the growth of endogenous cells and exogenous cells. In addition, in order to avoid the generation of ROS to hinder nerve regeneration during the treatment process, some functional nanoparticles can be added into the hydrogel to achieve the purpose of inhibiting the damage and promoting the regeneration [176, 177].

**Fig. 16:**
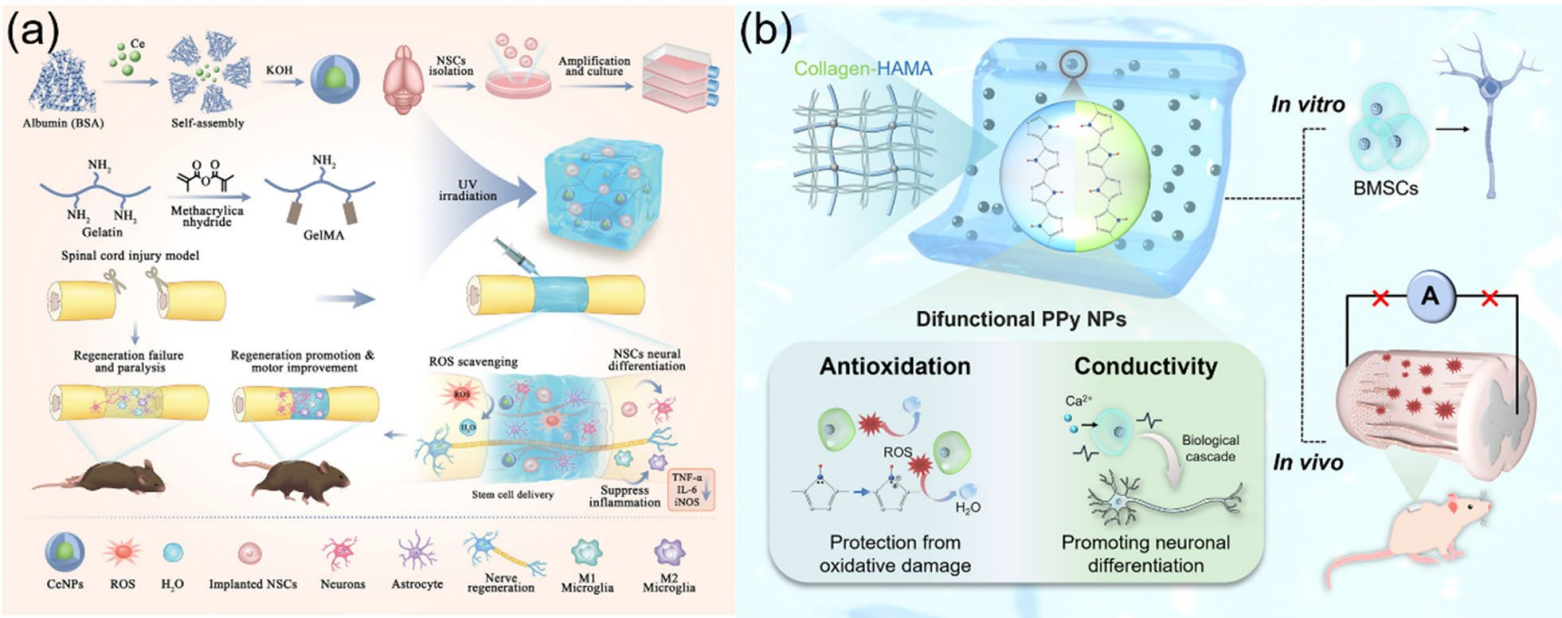
NP-functionalized bioactive hydrogels for SCI repair: **a** CeNP-Gel hydrogel for repairing SCI. Reprinted from Ref. [181], Copyright 2021, Wiley–VCH. **b** PPy NP-embedded collagen-HAMA hybrid hydrogel for in vivo SCI repair. Reprinted from Ref. [188], Copyright 2021, American Chemical Society

Nanozyme is a nano-biological material with enzyme-like activity, which has excellent application advantages in ROS scavengers [178–180]. Using the incubation strategy of BSA, Liu et al*.* successfully synthesized CeNPs with uniform and small size, and dispersed them in GelMA to obtain hydrogels with the ROS scavenging ability (CeNP-Gel) (Fig. 16a) [181]. This injectable hydrogel system could effectively induce the integration and differentiation of NSCs, increasing the survival rate of cells by about 3.5 times.

MnO_2_ NPs can catalyze the decomposition of H_2_O_2_ into H_2_O and O_2_ in the tumor microenvironment [182]. In the work of Li et al*.*, albumin was used as a biotemplate to biomimetically mineralize MnO_2_ NPs with good biocompatibility, which was dispersed in PPFLMLLKGSTR peptide-modified HA to obtain a bioactive hydrogel with good cell adhesion and neural tissue bridging ability [183]. Experiments have shown that HA can effectively inhibit the formation of neurotic scars. The incorporation of MnO_2_ NPs induced the formation of longer nerve fibers and alleviated the generation of glial fibrillary acidic protein (GFAP)-positive astrocytes, which can be excreted by circulating metabolism. CD31 labeling showed that the hydrogel system with MnO_2_ NPs showed better angiogenesis after 28 days of surgery, suggesting that MnO_2_ NPs have a good synergistic effect in inhibiting the glial scar formation and promoting the nerve fiber regeneration.

Bifunctional PPy with good conductivity and oxidation resistance, as a conductive polymer with good biocompatibility, can be used as a conductive scaffold for promoting the nerve tissue regeneration [184–187]. Wu et al*.* prepared PPy NPs with good dispersity based on a water-soluble PVA/iron ion system. The introduction of PPy NPs into the HA-collagen system successfully obtained conductive nanohydrogels with abundant cell adhesion sites (Fig. 16b) [188]. The incorporation of PPy NPs not only inhibited the increase of ROS, but also protected BMSCs from oxidative damage. In addition, the hydrogel promoted the transmission of intercellular electrical signals (ES) and external ES to BMSCs through its excellent electrical conductivity, and further promoted the neuronal differentiation of BMSCs through the PI3K/Akt and MAPK signaling pathways to achieve enhanced tissue repair capabilities.

Similarly, Zhang et al*.* used HA/collagen hydrogel as a substrate to endow it with good magnetoelectric capability by decorating the hydrogel with Fe_3_O_4_@BaTiO_3_ NPs. The hybrid hydrogel exhibited enhanced spinal nerve repair in the presence of an applied pulsed magnetic field [189]. Separately, Gao and the colleagues investigated a hydrogel composed of CTS and HA, in which a conjugated complex of Au NPs and ursodeoxycholic acid (UDCA) was incorporated into the hydrogel to treat local damaged areas under the irradiation of 808 nm NIR light [190]. The study showed that in the center of the injured spinal cord, Au NP-UDCA in the injectable hydrogel heated up to generate heat under NIR light irradiation, and effectively inhibited the production of inflammatory cytokines by macrophages when the ambient temperature reached 40 °C. This thermogenic approach exerts a pronounced anti-inflammatory effect on the damaged sites.

#### 1DM-incorporated hydrogels for SCI

1DM-based functional hydrogels have been utilized for various fields due to their porous structure and enhanced mechanical properties. For the fabrication 1DM hydrogels, some nanoscale materials with 1D morphology such as polymer fibers, CNTs, protein nanofibers, and peptide nanofibers can serve as the precursors to improve the properties and functions of hydrogels, which exhibited great potential for the SCI repair in the last years.

Hydrogels reveal advantages for treating SCI by delivering drugs and genes to the injuried sites to promote the direct axon regeneration. Chew et al*.* reported the fabrication of 3D aligned nanofibrous hydrogels for controllable drug/gene delivery to treat SCI [191, 192]. In their work, aligned poly (ε-caprolactone-co-ethyl ethylene phosphate) (PCLEEP) nanofibers were fabricated into the as-prepared collagen hydrogel to form the composite hydrogels with ordered architecture. It was found that the fabricated PCLEEP/collagen hydrogels not only imitated the size and architecture of natural ECM, but also provided a versatile nanoplatform for the loading and delivery of drugs (such as neurotrophin-3) and genes (such as microRNA), allowing the regeneration of several axons in the process of SCI repair. In this biomimetic 3D architecture, both PCLEEP and collagen exhibited synergistic effects on the SCI repair, in which aligned PCLEEP mediated robust cell penetration in vivo and neurite infiltration, meanwhile collagen promoted the cell adhesion and growth.

**Fig. 17.**
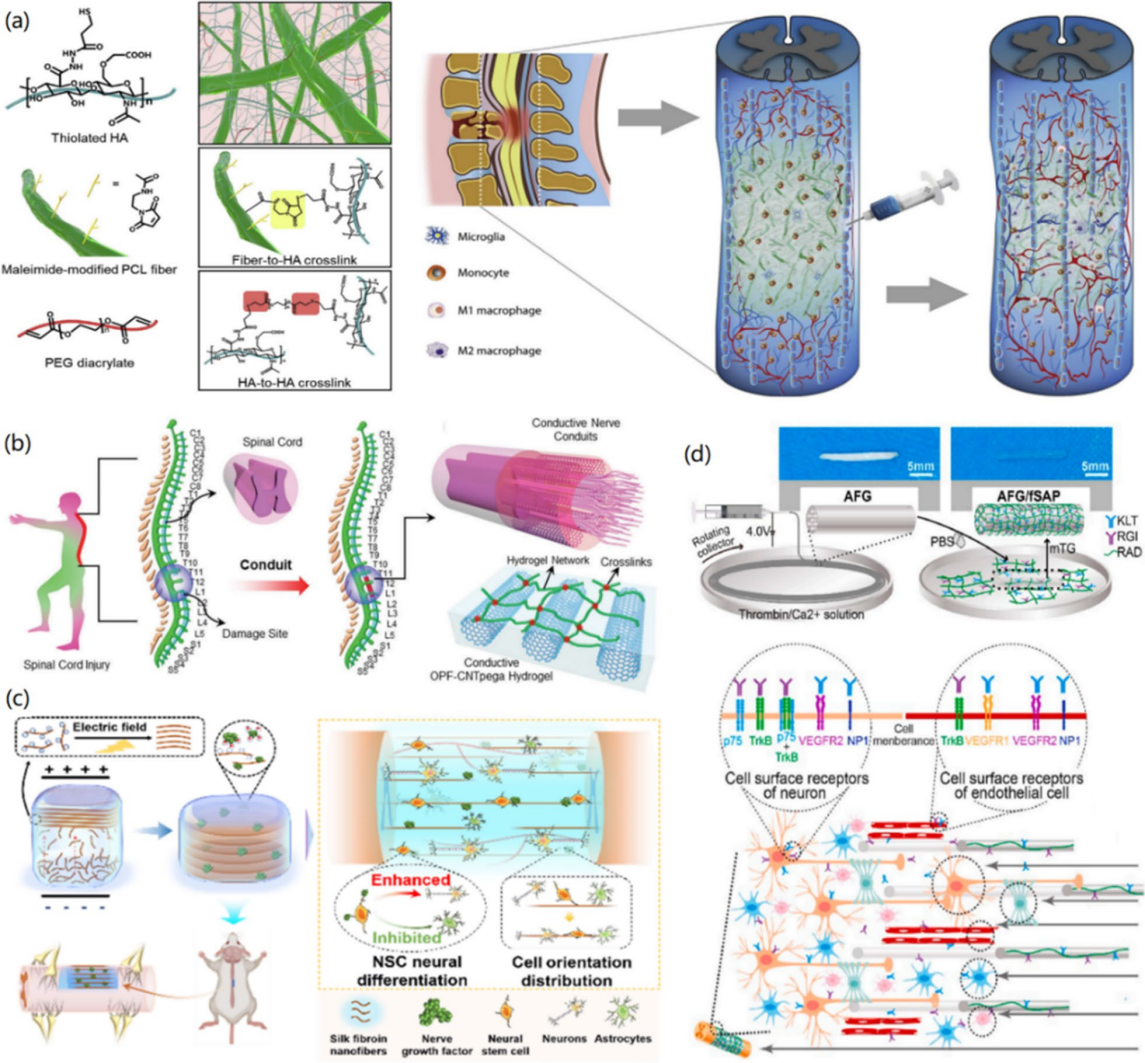
1DM-incorporated hydrogels for SCI repair applications: **a** electrospun MAL-PCL fiber-doped PEG-HA hydrogel for SCI repair. Reprinted from Ref. [193], Copyright 2020, Elsevier. **b** CNT-doped conductive PEG hydrogels for SCI repair. Reprinted from Ref. [195], Copyright 2018, Royal Society of Chemistry. **c** NGF-functionalized SFN hydrogels for scarless spinal cord repair. Reprinted from Ref. [200], Copyright 2022, American Chemical Society. **d** peptide nanofiber (PNF)-PEO AFG for spinal cord regeneration. Reprinted from Ref. [204], Copyright 2021, Elsevier

In another similar case, Li and co-workers demonstrated the fabrication of an injectable PCL-doped hyaluronic acid (HA) hydrogel for neural tissue repair and regeneration in spinal cord [193]. As shown in Fig. 17a, the design of hybrid hydrogel and interfacial bonding between each component are explained, in which the thiolated HA, maleimide-modified PCL fiber (MAL-PCL), and PEG diacrylate were mixed together to form the hydrogel. The cross-linking of HA to fiber and HA-HA stabilized the nanofibrous and network structure of the hydrogel. Ascribing to the addition of electrospun PCL fibers, it was possible to fabricate the composite hydrogel with a shear storage modulus of 210 Pa that similar to native spinal cord nervous tissue (50–600 Pa), and therefore the created hydrogels were potential candidates for repairing SCI. After injecting the composite hydrogel into the contused spinal cord, the designed hydrogels showed multi-functions for the SCI repair, including the inhabitation of collapse of spinal cord, the mediation of macrophage shift, and the promotion of cell invasion, blood formation, axon growth, as well as neuro-regeneration. Their study demonstrated a facile strategy to fabricate functional biocompatible hydrogels to mimic the spinal cord segment and microenvironment to facilitate the repair and regeneration of nervous tissues.

In the tissues, neurons are electrically responsive and can guide the transmit of electrical signals. Conductive hydrogels were beneficial for the creation of electrical signals in the tissues, and promoted the repair of SCI [142]. Due to their high mechanical properties and good conductivity, CNTs as scaffolds have been applied for improving the reconstruction of nerves by attracting stem cells to the injured tissues. Previously, Sang et al*.* reported the synthesis of conductive, thermos-responsive poly(n-isopropylacrylamide) (PNIPAAM) hydrogel by the modification with self-assembled CNTs [194]. The formed CNT-PNIPAAM hydrogel was injectable and highly conductive, could potentially promote the regeneration of nerve tissues and decrease the possibility of forming scar tissues in the process of SCI repair. In another case, Liu and co-workers fabricated conductive hydrogels with modulable conductivities for SCI repair by using PEG-modified CNTs (CNTpega) and oligo (poly (ethylene glycol) fumarate (OPF), as shown in Fig. 17b [195]. Due to their high conductivity, good biocompatibility, composited structure, the fabricated OPF-CNTpega hydrogel promoted the attachment, proliferation, and neuronal differentiation of PC12 cells. In addition, by producing the nerve conduits with the formed hydrogels through the injection molding technique, several types of nerve conduits have been prepared, which exhibited practical application in the SCI repair for guiding the regeneration of axons as the molds can be operated with tweezers easily. It was found that axons were grown across the injured sites and arrived the distal ends under the guidance of the hydrogel-based conductive conduits. This study provided a feasible biocompatible and conductive product that could be useful for clinical SCI repair.

In addition, 1D nanofibers with biocompatible properties, such as protein nanofibers and peptide nanofibers are highly useful for the repair of SCI, ascribing to their natural compatibility, bioactivity, and multiple groups with easy modification. Wang and co-workers have carried out a series of studies on the fabrication of aligned fibrin nanofiber hydrogels (AFG) with tailorable structures and functions for treating SCI [196–199]. In a typical case, they proved that the hierarchical AFG with soft stiffness and aligned ordered structure could promote the regeneration of nerves under the situations of in vitro and in vivo [198]. Induced by the fabricated AFG, white matter with consecutive, compact, and aligned nerve fibers have been regenerated, resulting in clear motor functional restoration of T12 SCI. In a very recent study, they further tailored the functions of the as-prepared AFG with N-Cadherin to enhance the functions of central nervous system and axon regeneration [199]. The modified AFG provided specific binding with NSCs, and could direct NSC functions and nerve regeneration, and therefore the designed hydrogels could carry exogenous NSC for repairing SCI through cell retention, immunomodulation, neuronal differentiation, and integration with inherent neurons. Besides silk fibroin nanofiber (SFN) hydrogels modified with nerve growth factor (NGF) have been presented for repairing scarless SCI [200]. As indicated in Fig. 17c, aligned SFN hydrogels were formed under the action of an applied electric field, which were then doped with NGF to form hybrid NGF-SFN hydrogels for enhanced neural differentiation and cell orientation and distribution. The created bioactive hydrogels provided a biomimetic microenvironment in vivo to guide the regeneration of scarless spinal cord, which showed similar microstructure to that of natural spinal cord, ascribing to synergistic effects of physical and biological properties.

Peptide nanofibers via the motif design and molecular self-assembly are versatile nanoscale building blocks for various materials science and biomedical applications, including the repair of SCI [201]. For instance, NSCs and neural progenitor cells have been embedded into the self-assembled peptide nanofiber (IKVAV/RGD hydrogels for nerve degeneration [202]. In addition, brain-derived neurotrophic factor and drugs (Chondroitinase ABC) [203] have been also applied for the functionalization of peptide nanofiber hydrogels for treating SCI. Recently, Man et al*.* presented a multi-modal delivery strategy for repairing SCI using AFG/functional self-assembling peptides (AFG/fSAP) composite hydrogel. The fabrication strategy of composite hydrogel and the SCI repair mechanism are shown in Fig. 17d. The using of the AFG/fSAP for rat SCI repair indicated that the hydrogels could improve the motor function recovery, facilitate the regrowth and angiogenesis of axon, guide the migration of astrocytic, and promote the remyelination [204]. In another study, Cao et al*.* prepared a hierarchically AFG with both aligned nanostructures and low elasticity, which can effectively promote nerve fiber regeneration in a rodent SCI model. In the follow-up application, AFG was also explored in the repair of canine lumbar 2-segment hemisection spinal cord injury. The results showed that after AFG implantation, its nanometer-to-millimeter-scale hierarchical arrangement endowed it with a unique guiding effect, enabling axonal re-growth in an oriented pattern connecting rostral and caudal stumps. This can significantly improve the recovery of motor function in dogs with SCI [197].Although these hydrogel materials revealed relative advantages and performances on SCI repair, the mechanisms on how the single components regulated the bioprocesses of SCI should be further investigated. In addition, more efforts on how to improve the nanofiber hydrogels for clinical applications should be considered seriously.

#### 2DM-incorporated hydrogels for SCI

2DMs, such as MoS_2_, GO, rGO, MXene, and MOFs have been widely used for the synthesis of various functional bioactive materials. 2DMs are potential candidates for the SCI repair because of their large specific surface area, good biocompatibility, easy modification, and potential conductivity, and catalytic activity [205].

**Fig. 18.**
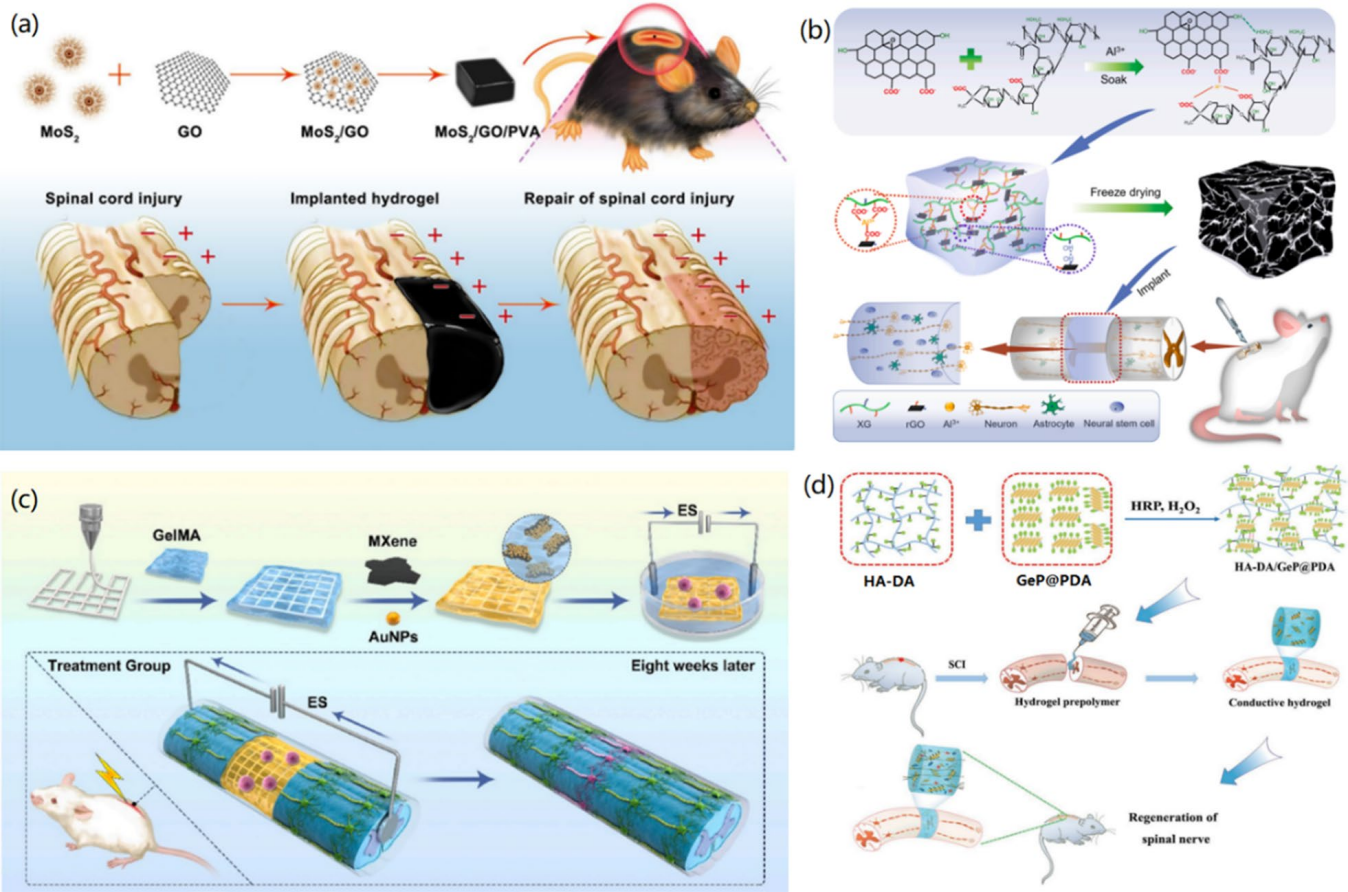
2DM-incorporated hydrogels for SCI repair applications: **a** MoS_2_/GO/PVA hydrogel for repairing SCI. Reprinted from Ref. [207], Copyright 2022, Springer Nature. **b** rGO/XG gel for repairing SCI. Reprinted from Ref. [209], Copyright 2022, Elsevier. **c** MXene and AuNPs-modified GelMA hydrogel for the recovery of SCI. Reprinted from Ref. [212], Copyright 2023, Elsevier. **d** 2D GeP@PDA-doped HA-DA hydrogel for enhanced repair of SCI. Reprinted from Ref. [11], Copyright 2021, Wiley–VCH

Marques and co-workers have raised a question “Is graphene shortening the path toward spinal cord regeneration?” in a recent review [206]. By detailed analysis on a lot of studies that using graphene-based materials for SCI, they have concluded that graphene-based materials play important roles in developing complementary SCI therapeutic approaches and promoting neuroregeneration from enhanced neural cell-material interactions. In addition, they proposed that the using of graphene materials could shorten the way to achieve in clinical translation. Based on the unique physical and chemical properties of MoS_2_ and GO, Chen et al*.* demonstrated the fabrication strategy of MoS_2_ and GO-functionalized PVA (MoS_2_/GO/PVA) composite hydrogels for repairing SCI [207]. As shown in Fig. 18a, MoS_2_/GO nanohybrids were firstly prepared by conjugating MoS_2_ onto the surface of GO nanosheets, which were then mixed with PVA to form composite hydrogels via repeatedly freezing and thawing. Due to the addition of MoS_2_ and GO, the created composite hydrogels exhibited good suppleness, high mechanical properties, and high electrical conductivity. After injecting the hydrogels into the SCI part, the differentiation of NSCs into neuron cells and scavenged ROS were induced. Meanwhile, the using of the composite hydrogels inhibited the differentiation of M1 and active M2 macrophage, which improved inflammatory cytokines effectively. In this case, the unique properties, such as the high conductivity of MoS_2_ and the good mechanical properties of GO, extended the potential of PVA hydrogels for repairing SCI.

In another case, Zhang and co-workers synthesized GO-functionalized diacerein-terminated PEG hydrogels by using the strong interactions between GO and diacerein, and further applied the formed hydrogels for repairing SCI [208]. They also proved that the electric conductivity and 3D porous structure of the hydrogels accelerated functional formation and neural activity in the neural network by mediating the migration of neural system cells and the remyelination of axons. Although the GO-based hydrogels have been presented as effective candidates for repairing SCI, the understanding on the signaling pathways in the process of repair should be studied further at the molecular level.

Although GO has satisfied biocompatibility for the SCI repair, its low electrical conductivity could be improved by the reduction of GO into reduced GO (rGO) for enhanced neuroregeneration. In a typical study, Xue et al*.* demonstrated the design and fabrication of a electroconductive and highly porous rGO/xanthan gum (rGO/XG) hydrogel for repairing SCI, as shown in Fig. 18b. Benefit by the using of rGO, the formed hydrogel exhibited high electroconductivity, which promoted the transmission of electrical signals, guided the ordered growth of regenerated nerve fibers, and inhibited the formation of glial scars [209]. In addition, the in vivo tests with rats indicated that the injection of hydrogels to SCI parts mediated the restore of the motor function of rats. After the reduction to graphene, GO reveals lower biocompatibility, which could decrease the functions for cell adhesion and growth. Therefore, the using of highly biocompatible components with graphene for the fabrication of composite hydrogels is necessary. For instance, the cross-linking graphene with collagen [209, 210] and silk proteins [211] for the synthesis of biocompatible graphene-based hydrogels for repairing SCI have been reported.

Besides MoS_2_ and graphene materials, other 2DM-bade hydrogels have also been utilized for the SCI repair applications, although very limited studies have been reported. For instance, Kong and co-workers reported the design and synthesis of a multifunctional hydrogels by modifying the GelMA hydrogels with MXene and AuNPs for SCI repair [212]. As indicated in Fig. 18c, GelMA hydrogels were first prepared and MXene and AuNPs were then added into the as-prepared hydrogels to form MAu-GelMA composite hydrogels. After loading NSCs into the formed composite hydrogels, the hydrogels exhibited combined treatment for repairing SCI. First, the loaded NSCs can promote the recovery of SCI by the injection of hydrogels into the injury parts. On the other hand, the good electrical conductivity of both MXene and AuNPs promoted NSC differentiation and myelin regeneration, resulting in functional recovery of SCI. The combined therapy with functional hydrogels could be an effective strategy for repairing SCI. In another study, conductive and biodegradable germanium phosphide (GeP) nanosheets have been utilized for the modification of hyaluronic acid-dopamine (HA-DA) hydrogels for enhanced SCI repair, as show in Fig. 18d. The in vitro experiments indicated that the formed HA-DA/GeP@PDA hydrogels accelerated the differentiation of NSCs into neurons and the in vivo tests with rat models proved further that the hydrogels improved the recovery of locomotor function of rats effectively [11]. In addition, other 2DMs, such as ZIF-based hydrogels are also beneficial for repairing SCI, ascribing to their versatile molecular design, tailorable functions, high biocompatibility, and large potential in biomedicine and tissue engineering [213].

### Clinical development of bioactive hydrogels

It is well known that currently no effective medical treatments can be utilized for reversing the damage of SCI as the repair of SCI is a complex process that related to chemical, physical, and biological aspects. The treating performance of traditional methods, such as surgy, drug therapy, and rehabilitation could result in limited effects on the repair of SCI [206]. Previous small animal test and clinical studies indicated that the using of neuroprotective drugs, NSCs, and neuromodulatory stimulations are beneficial for promoting the neuroregeneration in the SCI area, which has guided the development direction of clinical treating SCI.

3D hydrogels with high bioactivity and biocompatibility can act as excellent vehicles for delivering NSCs and drugs for SCI treatment, and are highly potential for clinical development of SCI repair. In the above introduction and discussion, we find that bioactive hydrogels have been widely used for preliminary lab study on repairing SCI, and in most of the cases, the designed bioactive hydrogels have exhibited great performance for treating SCI, not only in the in vitro cell test, but also in the in vivo tests with small animals such as rats. However, very limited efforts have been carried out for using bioactive hydrogels for real clinical human trials. A few factors could be crucial for affecting the clinical development of bioactive hydrogels.

The bioactivity and biocompatibility, as well as their physical properties of bioactive hydrogels are unknown for large animals currently. The pre-fabricated hydrogels can mediate the regeneration of axons, but reveal high risk to damage the spared neuro tissues; the injectable hydrogels are versatile and less-invasive to fill in the irregularly shaped lesion, but they will inhibit the axonal regeneration [14]. In addition, the conditions in large animals are much complicated than that of rats, the chemical and biological reactions in both cases may have big differences [214].

The clinical trials need effective drugs usually. For the SCI drug therapy with bioactive hydrogels that loaded drugs, the cost will be very high. It is known that average time and cost for developing a useful drug that can be approved by the FDA are about 10 years and 1 billion dollars. At present, it is not easy to find a highly effective and approved drug for repairing SCI. Previously, a bioactive drug, anti-NogoA, has been testified as one potential candidate for the treatment of SCI with a phase I clinical trial [215]. It was still very far for the real clinical success.

Besides, there are many and complex mechanisms of bioactive hydrogels towards the repair of SCI. As discussed in 2.1.1, there are four common mechanisms mentioned in the section, and the action mechanisms of bioactive hydrogels for repairing SCI are also complex, and in some cases, multiple actions are responsible for SCI repair. Without fully clear understanding these mechanisms, it is hard to apply hydrogels for clinical trials really.

## Conclusion and perspectives

In summary, we presented a comprehensive review on the design, synthesis, functional regulation of bioactive hydrogels for repairing SCI. Based on the above introductions and discussions, several key conclusions are given. Firstly, the development of materials science and nanotechnology provided good opportunities for the SCI repair studies. Various biomaterials are widely utilized for the preliminary and pre-clinical studies of SCI repairing, in which bioactive hydrogels showed some advantages such as 3D porous structure, high biocompatibility, injectability, easy operation, and similar properties to ECM. Secondly, bioactive hydrogels can be fabricated through chemical and physical cross-linking of various biomolecules, including DNA, proteins, peptides, biomass polysaccharides, and other types of biopolymers. These natural and synthetic biomolecules provided potential bioactivity and biofunctions for the synthesized hydrogels for repairing SCI. In addition, the loading of NSCs, drugs, GFs, and molecular active factors into hydrogels promoted the multi-functions of the composite hydrogels, further improving the repair efficiency of injected hydrogels in SCI sites. Thirdly, we demonstrated various methods for regulating the biological properties of bioactive hydrogels, such as the cell biocompatibility, self-healing, antibacterial, bio-adhesion, biodegradation, and others, which played crucial roles in promoting the neuroregeneration in the SCI sites and mediating the recovery of motor function. Finally, in the SCI repair studies, the functions of bioactive hydrogels can be further regulated by introducing drugs/GFs, NPs, stimuli-responsive polymers, 1D materials, and 2D materials. These efforts provided more electrical, optical, thermal, and enzymatic functions or properties for the fabricated bioactive hydrogels, greatly inspiring the SCI repair applications from preliminary to clinical studies.

Bioactive hydrogels have shown great potential for the repair of SCI in the last years, according to the above analysis. Here we would like to provide our viewpoints on the further development on using bioactive hydrogels to treat SCI. First, more efforts should be done to understand the signaling pathways and corresponding repair mechanism by active hydrogels in the processes of SCI repairing. The theoretical studies can guide the design and synthesis of hydrogels with specific properties and functions for SCI. Second, there is challenge on the embedding and differentiation of stem cells in hydrogels, which could be the most effective ways to promote the intrathecal transplantation and neuroregeneration. Therefore, new techniques on the loading of stem cells into hydrogels and the creation of suitable cell proliferation conditions should be developed. Third, in the term of material design, some functional 2D materials, such as black phosphorus, MOFs, COFs, and MXenes could be combined with bioactive hydrogels to provide catalytic, enzymatic, and electrical functions of hydrogels, inducing specific applications in repairing SCI. Fourth, new fabrication techniques, such as 3D and 4D printing, could be developed to fabricate hydrogels with hierarchical structures that similar to natural human spinal cord. Meanwhile, the created hydrogels should maintain good injection ability and flexibility to fill in the injured sites. Fifth, the treatment of SCI with bioactive hydrogels should be combined with advanced therapy techniques, such as the biosensing, bioimaging, and physical diagnostics (for instance MRI and CT), for which functional NPs and molecular imaging agents should be applied. Final, we suggest that it is necessary to develop safe and effective drugs for treating SCI with further clinical trials, as currently nearly all the SCI repairing studies were focusing on pre-clinical studies with traditional drugs and GFs.

## Data Availability

The data shown in this article can be obtained by request.
